# Vertical distribution and seasonal dynamics of planktonic cyanobacteria communities in a water column of deep mesotrophic Lake Geneva

**DOI:** 10.3389/fmicb.2023.1295193

**Published:** 2023-12-15

**Authors:** Anna Carratalà, Coralie Chappelier, Oliver Selmoni, Annie S. Guillaume, Hannah E. Chmiel, Natacha Pasche, Charlotte Weil, Tamar Kohn, Stéphane Joost

**Affiliations:** ^1^Environmental Chemistry Laboratory, ENAC, École Polytechnique Fédérale de Lausanne (EPFL), Lausanne, Switzerland; ^2^Department of Embryology, Department of Plant Biology, Carnegie Institution for Science, Washington, DC, United States; ^3^Laboratory for Biological Geochemistry (LGB), Geospatial Molecular Epidemiology Group (GEOME), ENAC Faculty, École Polytechnique Fédérale de Lausanne (EPFL), Lausanne, Switzerland; ^4^Eusserthal Ecosystem Research Station (EERES), Institute for Environmental Sciences (iES), University of Kaiserslautern-Landau, Landau, Germany; ^5^Limnology Center, École Polytechnique Fédérale de Lausanne (EPFL), Lausanne, Switzerland; ^6^ENAC-IT4R, École Polytechnique Fédérale de Lausanne (EPFL), Lausanne, Switzerland

**Keywords:** cyanobacteria, bacterioplankton, lake, column, *Planktothrix*

## Abstract

**Background:**

Temperate subalpine lakes recovering from eutrophication in central Europe are experiencing harmful blooms due to the proliferation of *Planktothrix rubescens*, a potentially toxic cyanobacteria. To optimize the management of cyanobacteria blooms there is the need to better comprehend the combination of factors influencing the diversity and dominance of cyanobacteria and their impact on the lake’s ecology. The goal of this study was to characterize the diversity and seasonal dynamics of cyanobacteria communities found in a water column of Lake Geneva, as well as the associated changes on bacterioplankton abundance and composition.

**Methods:**

We used 16S rRNA amplicon high throughput sequencing on more than 200 water samples collected from surface to 100 meters deep monthly over 18 months. Bacterioplankton abundance was determined by quantitative PCR and PICRUSt predictions were used to explore the functional pathways present in the community and to calculate functional diversity indices.

**Results:**

The obtained results confirmed that the most dominant cyanobacteria in Lake Geneva during autumn and winter was *Planktothrix* (corresponding to *P. rubescens*). Our data also showed an unexpectedly high relative abundance of picocyanobacterial genus *Cyanobium,* particularly during summertime. Multidimensional scaling of Bray Curtis dissimilarity revealed that the dominance of *P. rubescens* was coincident with a shift in the bacterioplankton community composition and a significant decline in bacterioplankton abundance, as well as a temporary reduction in the taxonomic and PICRUSt2 predicted functional diversity.

**Conclusion:**

Overall, this study expands our fundamental understanding of the seasonal dynamics of cyanobacteria communities along a vertical column in Lake Geneva and the ecology of *P. rubescens*, ultimately contributing to improve our preparedness against the potential occurrence of toxic blooms in the largest lake of western Europe.

## 1 Introduction

Bacterial communities rapidly respond to numerous biological and environmental factors, such as protist grazing, viral infections, predatory bacteria, temperature, salinity, or nutrient concentrations (Fenchel, 2002; Berdjeb et al., 2011; Parvathi et al., 2014; Ezzedine et al., 2020). It is therefore likely that environmental changes will have a significant impact on the composition and evolution of bacteria communities in freshwater environments, both directly and indirectly (Weckström et al., 2016; Kraemer et al., 2021).

Research suggests that certain bacteria, such as cyanobacteria, may be favored by environmental conditions imposed by global warming, water eutrophication and human development (Huisman et al., 2018). Cyanobacteria are of notable importance, as some strains can produce toxins harmful to humans and other mammals and can cause large harmful cyanobacteria blooms (HAB) that deteriorate water quality, sometimes leading to widespread death of other aquatic species (Paerl and Paul, 2012; Paerl and Otten, 2013; Chorus and Welker, 2021). Therefore, it is important to accurately identify the combinations of environmental and biological factors that influence bacterial community dynamics, particularly with regards to cyanobacteria that can represent a threat to public health (Jacquet et al., 2005).

The impact of cyanobacteria proliferation on microbial community composition and functions remains elusive. While recent work has noted negative impacts of cyanobacteria blooms on zooplankton and phytoplankton diversity and function (Jia et al., 2017; Krztoń et al., 2019; Amorim and Moura, 2021), other studies have shown that cyanobacteria may cause both increases or decreases in diversity and function of aquatic communities (Sukenik et al., 2015). Less is known about the effects of cyanobacteria on bacterioplankton communities and their functions in mesotrophic and oligotrophic environments. However, studies available to date, developed mostly in eutrophic lakes and reservoirs, have noted an impact of cyanobacteria such as *Microcystis aeruginosa* on bacterioplankton composition and succession (Zheng et al., 2008; Guedes et al., 2018; Mankiewicz-Boczek and Font-Nájera, 2022).

Cyanobacteria from the *Planktothrix* genus play a crucial role in lakes where oligotrophic conditions are being restored through corrective measures (Jacquet et al., 2005; Ernst et al., 2009; Fernández Castro et al., 2021). Two examples of such lakes occur in Switzerland, where both Lake Zurich and Lake Geneva have recovered from strong eutrophication periods in the 1970s (Posch et al., 2012; Fernández Castro et al., 2021). In these restored lakes, *Planktothrix rubescens* has the potential to rapidly multiply and emerge as the dominant species among the phytoplankton communities, as bacteria of this genus are well-adapted to low light environments, regulating their position in the water column using gas vesicles and undergoing physiological adaptations (Walsby et al., 2004, 2006; Walsby, 2005; Yankova et al., 2016; Gallina et al., 2017). *Planktothrix rubescens* in Lake Zurich have been shown to accumulate as a persistent thin layer in the metalimnion during the stratified season (typically between May–October), which then disappears in autumn with the deepening of the mixed layer (Fernández Castro et al., 2021).

As the largest and deepest lake in western Europe, with a volume of 89 km^3^ and a surface area of 580 km^2^, Lake Geneva is of major importance for the region as a source of drinking water for 900,000 people, as well as for tourism development and fishing (Gallina et al., 2017). This monomictic lake (mixed from top to bottom during one period per year) experiences thermal stratification from spring to early winter (Minaudo et al., 2021).

Research suggests that the abundance of cyanobacteria could increase in Lake Geneva by 34% by the end of this century under future climatic conditions, potentially inducing significant changes in the microalgal composition and posing a threat for water quality (Paerl and Otten, 2013; Huisman et al., 2018; Amorim and Moura, 2021). *Planktothrix rubescens* has been shown to be a dominant cyanobacterial species in the water column of Lake Geneva since the early 2000’s (Jacquet et al., 2005). However, despite extensive monitoring since 1957 as part of the international water protection program led by the “Commission Internationale pour la Protection des Eaux du Léman” (CIPEL; International Commission for the Protection of Lake Geneva Waters), the temporal dynamics and diversity of cyanobacteria, as well as their impact on microbial diversity and functions in Lake Geneva, remain unknown.

In this study we aimed to unravel the dynamics of cyanobacteria throughout the water column of Lake Geneva and assess its impacts on the microbial community of the lake. Our hypothesis was that *P. rubescens* is the most dominant cyanobacteria in Lake Geneva and that in nutrient-poor lakes outcompetes other bacteria communities reducing bacterioplankton abundance, diversity and function (Walsby, 2005). Our specific goals were to (i) identify the main cyanobacteria genera present in a water column of Lake Geneva throughout the year, (ii) to characterize their seasonal dynamics and main environmental drivers and finally, (iii) to determine changes in the bacterioplankton community that may be associated with the dominance of *P. rubescens*. The dataset obtained in this study and the initial findings reported here mark an important stride towards improving our ability to make precise predictions regarding the potential effect of climate changes on the proliferation of the diverse cyanobacteria groups in Lake Geneva and the impact on the lake’s ecosystem services.

## 2 Materials and methods

### 2.1 Study location, sample methods and processing

**Figure 1:**
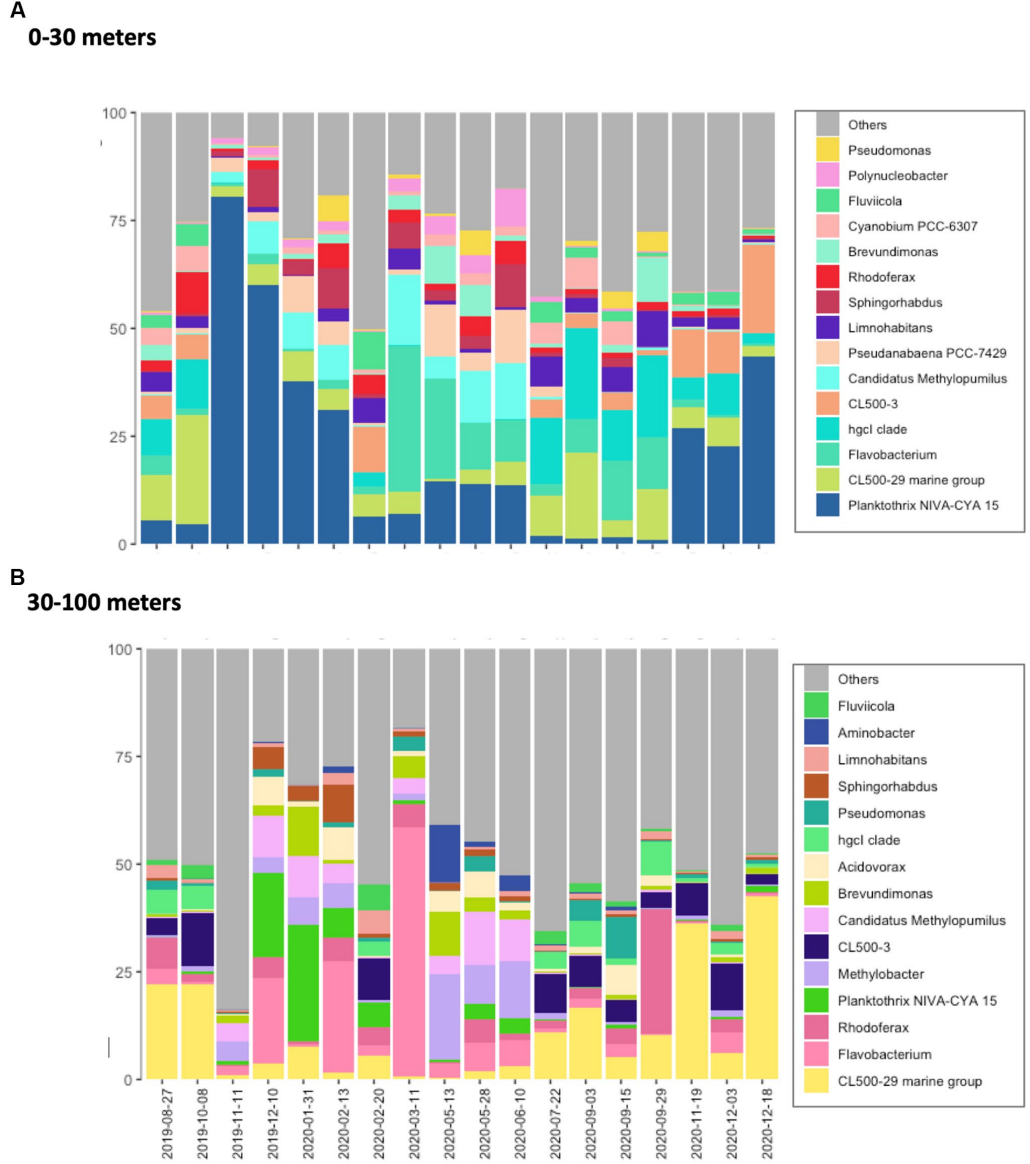
Bacterioplankton community composition in Lake Geneva. Main bacteria genus identified in the **(A)** top (0–30 m depth) and **(B)** bottom (30–100 m depth) layers of the studied water column. Compositional data was obtained by averaging the relative abundances determined in 155 and 58 water samples collected at different depths within the top and bottom layer, respectively.

Water samples were collected in Lake Geneva from the scientific platform LéXPLORE (46°30′0.819″ N, 6°39′39.007″ E), located 570 meters offshore from the town of Pully, Switzerland (Wüest et al., 2021a,b). Lake Geneva is a deep perialpine lake (maximum depth is 309 meters) located in the north of the Alps at 372 meters above the sea level (Supplementary Figure S1). We conducted monthly sampling campaigns between August 2019 and December 2020, collecting and processing a total of 218 samples over 18 dates. The sampling campaigns planned during the spring months of 2020 coincided with lockdown orders due to the COVID-19 pandemic such that fewer samples were collected during that period. During each sampling campaign, two-liter samples were collected using a Niskin bottle (HydroBios Kiel) and a 750 W electric winch (KC Denmark A/S). The maximum depth under the floating platform is approximately 110 meters, and samples were taken at 12 distinct depths (0, 2.5, 5, 10, 15, 20, 25, 30, 50, 60, 80, and 100 meters) (Figure 1). Upon collection, water samples were stored in polycarbonate bottles, transferred to a laboratory at EPFL, and kept at 4°C until they were filtered within the next 24 h. One liter per sample collected between 0 and 50 m depth, and two liters per sample from 50 to 100 m depth, were filtered through 0.45 μm filters (Millipore) using an electrostatic pump. Filters were kept frozen at −20°C until nucleic acid extractions were performed.

### 2.2 Water analysis and compilation of meteorological data

The physicochemical properties of the water column were monitored during each sampling campaign using a multiparameter probe (Sea & Sun Technology GmbH), which determined vertical gradients of conductivity, temperature, pressure, pH, chlorophyll a, oxygen saturation, dissolved oxygen concentration and turbidity (data shown in Supplementary Figure S2). Nutrient concentrations [i.e., total phosphorous (TP), total nitrogen (TN), total dissolved phosphorous (TDN), total dissolved nitrogen (TDN), dissolved inorganic phosphorous (DIN), and dissolved inorganic nitrogen (DIN)] were determined from a subset of the water samples at the Department of Surface Waters of the Swiss Federal Institute of Aquatic Sciences (Eawag) following standard protocols (Supplementary Figure S3). Meteorological data (air temperature, precipitation, wind speed and irradiance) were pulled from the nearby Pully weather station for each sampling day, retrieved from the Swiss Federal Office of Meteorology and Climatology (Meteoswiss) on July 27th, 2022.

### 2.3 DNA extraction and determination of 16 s gene abundances by quantitative PCR

Nucleic acid extractions were performed using the DNeasy^®^ PowerWater^®^ kit according to the manufacturer’s instructions with minor modifications (nucleic acids eluted in 60 μL of elution buffer). To monitor the abundance of bacterioplankton biomass in the samples, quantitative real-time PCR assays (qPCR) were performed using the Femto Bacterial DNA quantification kit (Zymo Research Corporation), which includes primers and standards, following the thermocycling conditions recommended by the manufacturer. The average efficiency of all the standard curves was 0.92 and the average *R*^2^ was 0.98. All qPCR data were retrieved using the thermocycler Mic Real-Time PCR System (LabGene Scientific, SA).

### 2.4 MiSeq sequencing of 16S rRNA amplicons and data processing

Prior to the DNA library preparation, 2 μL of the extracted nucleic acids from each sample were quantified using a Qubit^®^ fluorometer (Thermo Fisher scientific United States) and the Qubit^™^ 1x dsDNA HS Assay kit (Thermo Fisher scientific United States). Samples were sent to Fasteris (Genesupport, SA. Geneva) for library preparation and next generation sequencing using MiSeq (Illumina). Libraries were prepared following the Metafast protocol, an in-house procedure of Fasteris (Genesupport, SA. Geneva). To obtain the library, PCR targeting the V3–V4 region of the 16S gene of bacteria were conducted for every sample. The primers used were 341F (5′-CCTACGGGNGGCWGCAG-3′) and 805R (5′-GACTACHVGGGTATCTAATCC-3′) (Klindworth et al., 2013). The obtained sequences were sorted using an in-house script property of Fasteris, trimmed using Trimmomatic and joined using the ea-utils obtaining FastQ scores for each sample. Finally, the sequences were classified as operational taxonomic units (OTUs) using the Burrows-Wheeler Alignment Tool and the taxonomically clustering was performed using Silva rRNA database based on 97% sequence similarity.

### 2.5 Data analysis

The data analysis and plots were conducted in RStudio (R version 4.1.3) (R Core Team, 2012). The qPCR results were visualized using the ggplot2 and lattice packages (Grothendieck, 2008; Darrin and Bryan, 2021). R functions levelplot and panel. 2dsmoother were used to plot smooth approximations of 16S rRNA abundance, environmental data and Chao1 over dates and depths using the method of locally estimated scatterplot smoothing (LOESS). An analysis of variance (ANOVA) was performed to determine the statistical significance of differences in 16S rRNA abundances between different layers and seasons. The microeco package was used for downstream analysis of the sequencing data (Liu et al., 2021). Mitochondria and chloroplast sequences were removed from the dataset, and Chao1 index was selected to assess alpha diversity. The spatiotemporal abundance of specific bacterial genera was plotted using the raster and gstat R packages (Hijmans and van Etten, 2015). The prediction of metabolic pathways and functional profiles of the identified communities was determined using QIIME2 (version 2021.2) and PICRUSt2 (version 2.4.0) (Langille et al., 2013; Bolyen et al., 2019; Douglas et al., 2020). The OTUs underwent closed reference picking in QIIME2 using a database that was trained on Greengenes version 13.5, employing a 97% identity cluster. The resulting feature table was exported as a biom format file, normalized by the predicted 16S rRNA copy number and employed in PICRUSt2 for predicting counts of metabolic functions. This prediction was accomplished by referencing the Kyoto Encyclopedia of Genes and Genome (KEGG) Orthology (KO) Database (Kanehisa and Goto, 2000). The accuracy of metagenomic prediction was assessed using the Nearest Sequenced Taxon Index (NSTI) values as previously described (Langille et al., 2013). Chao1 indices were calculated to assess functional diversity in the samples.

The Kruskal–Wallis rank sum test was employed to determine the significance of differences in alpha diversity and taxon abundance among different groups for both taxonomic and functional diversity. Bray–Curtis dissimilarity was used to measure the dissimilarities between pairs of communities, and non-metric multi-dimensional scaling (NMDS) was used to visualize differences among communities. The relative abundances of important genera were correlated with environmental factors using the Spearman correlation method. Prior to calculating Spearman correlations, autocorrelation between environmental variables was assessed using multicollinearity analysis, and variance inflation factors (VIF) were calculated using the R package car (Fox and Weisberg, 2019). Variables with VIF ≥5 were excluded from each pair, where the variable retained was based on its assumed biological relevance (James et al., 2019). Of the 22 initial measured variables included in the correlation analyses, 10 variables were retained. To identify significant biomarker functions for the productive and unproductive zones, as well as for each season at both taxonomic and functional level, linear discriminant analyses (LDA) with differential abundance tests implemented in LEfSe were applied (Segata et al., 2011).

## 3 Results

### 3.1 Environmental characterization of the water column

The environmental variables and nutrient concentrations measured in this study clearly reflect the spatiotemporal variations of the local weather conditions and in the water column throughout the year due to the stratification and deep mixing of the lake (Supplementary Figures S2, S3). The water column appeared fully mixed from December to April (Supplementary Figure S2). During deep mixing, the water temperature reached its minimum at 6°C, while the highest water temperature was 22°C, which was recorded at the surface of the water column in September 2020. Nutrient measures were determined for a subset of the 218 samples collected in the study and showed higher concentrations in the unproductive layer than in the productive layer, particularly in winter and spring (Supplementary Figure S2). Measures of TN, TDN and DIN were higher in winter at the water surface and below 30 m across the sampling period. TN, TDN and DIN were, as expected, shown to be highly correlated (Pearson’s correlation index >0.75). The highest values of DIP were observed in winter in the homogenized water column reaching 5 mg/L. TDP and TP concentrations showed less variability in the water column across seasons, but concentrations were generally higher in the unproductive layer compared to the productive layer in every season.

### 3.2 Bacterioplankton community structure

From the 218 water samples collected in this study, 56 samples were taken in autumn, 34 in spring, 46 in summer and 82 in winter (two winter seasons were included in the study, which lasted 15 months). With regards to the physical state of the water column, 131 samples were collected during lake stratification and 81 during deep mixing. The sequencing of the collected samples produced almost 25 million of Bacteria sequences and a total of 12,748 OTUs were identified. On average each sample produced 115,621 reads and 731 OTUs.

**Figure 2:**
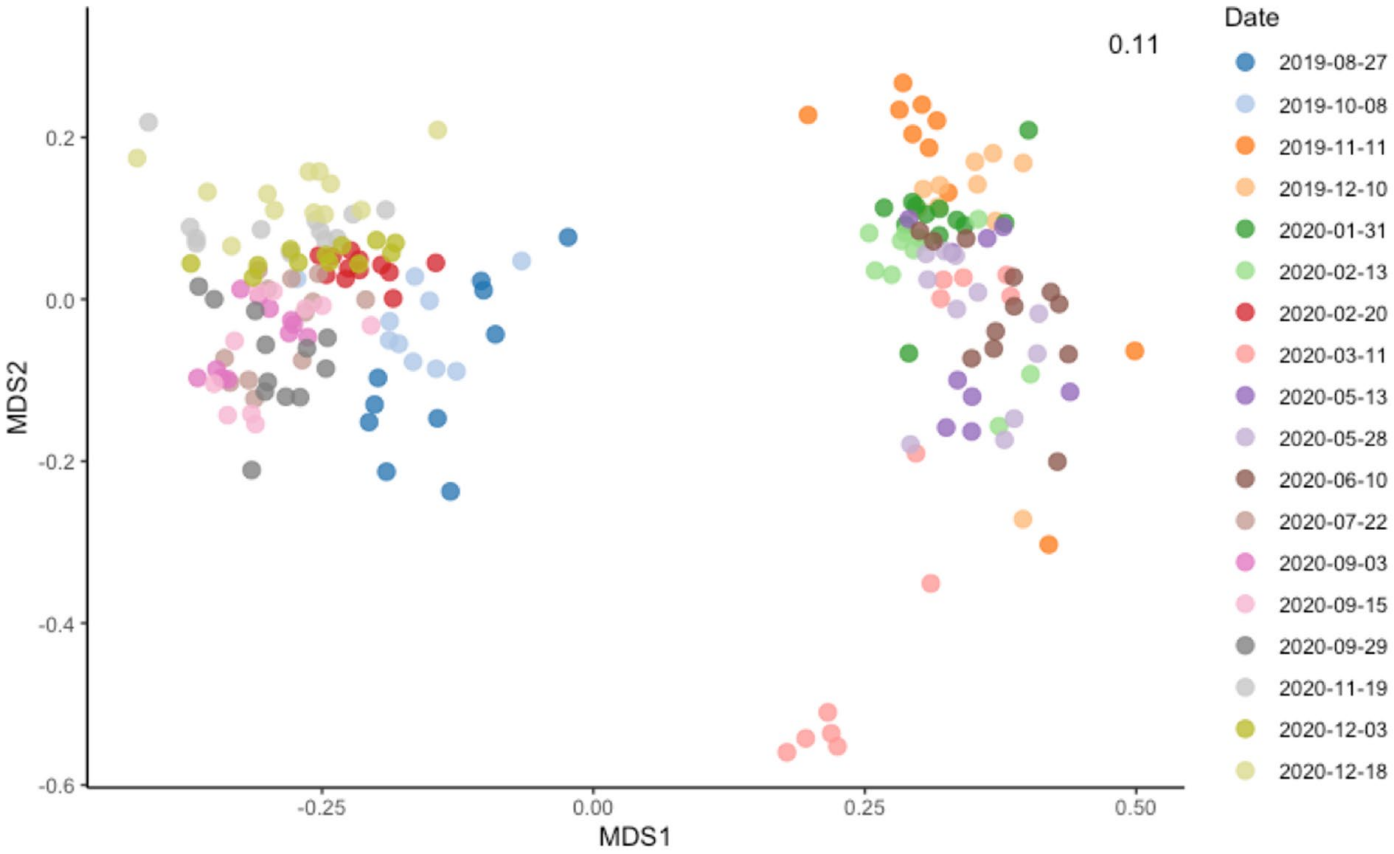
Non-metric multidimensional scaling (MDS) plot showing Bray–Curtis dissimilarity among samples. dbRDA ordinations of the bacteria communities obtained using Bray Curtis distance estimates and colored based on the sampling date.

The five most abundant bacteria phyla in Lake Geneva at the time of this study were *Proteobacteria*, *Actinobacteriota*, *Bacteroidota*, *Cyanobacteria* and *Planctomycetota*. These phyla accounted for more than 75% of the sequenced reads and were both present along the water column. The most abundant bacteria genera identified in the dataset were *CL500-29 marine group*, *Flavobacterium*, *Planktothrix*, *CL500-3*, *hgcl clade* and *Rhodoferax* (Figure 1). Most bacteria genera were observed both in the surface and the bottom layer (2,867), however certain groups appeared exclusively in the top or bottom layer (1,699 and 1,119 genera, respectively). Beta diversity analysis based on Bray–Curtis dissimilarity distances show that community dissimilarity was mostly driven by compositional changes occurring at specific dates (Figure 2; Supplementary Figure S4), with flare ups occurring from November 2019 to February 2020 and from March 2020 to June 2020.

Linear discriminant analysis (LDA) with LDA effect size analysis (LEfSe) allowed us to identify taxonomic biomarkers and functional pathways defined in the Kyoto Encyclopedia of Genes and Genomes (KEGG) that were significantly associated with the top and bottom layers of the water column. These results (Supplementary Figure S7) suggest that several cyanobacteria genera (*Planktothrix*, *Pseudoanabaena*, *Cyanobium* and *Aphanizomenon*) and the genus *Limnohabitans* were markers of the top layer of the studied column (LDA scores above 2.5) and consequently, PICRUSt2-predicted functions involving energy metabolism and photosynthesis were identified as biomarkers (LDA scores above 2). Conversely, the genera *Pedobacter*, *Undibacterium*, *Nitrospira* and *Methylobacter* were markers of the bottom layer (LDA scores from −2.5 to nearly −5). At the functional level, the bottom of the water column was enriched in pathways for the metabolism of aminoacids and carbohydrates, cell motility and transport, and the processing of environmental information (LDA scores below −2; Supplementary Figure S7).

### 3.3 Cyanobacteria diversity and spatiotemporal dynamics

**Figure 3:**
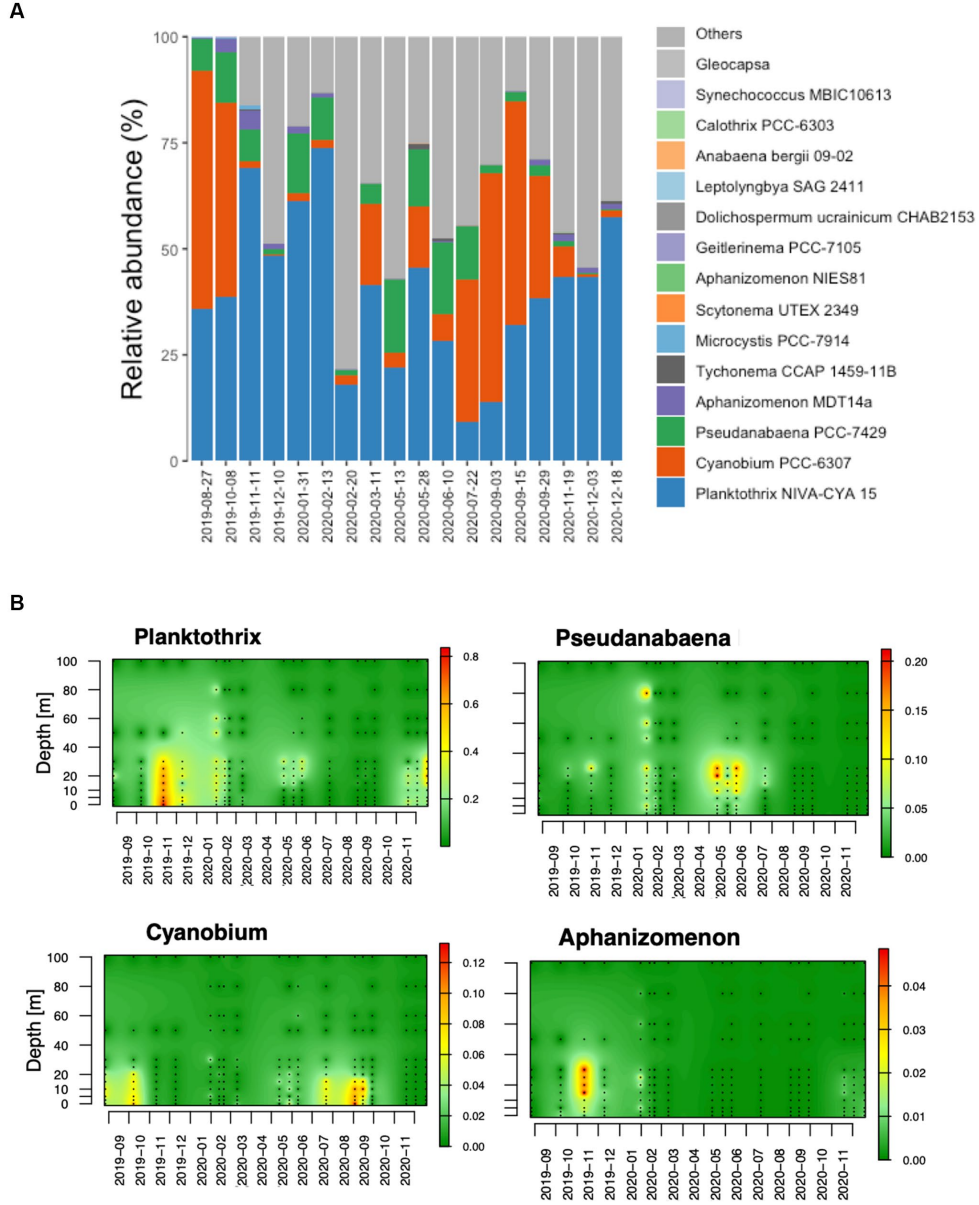
Cyanobacteria community composition in Lake Geneva. **(A)** Main cyanobacteria genus identified in the studied water column. The compositional data was obtained by averaging the relative abundance of cyanobacteria in 218 water samples. **(B)** The panel below shows the spatiotemporal abundance of genera *Planktothrix* and *Flavobacterium* as relative abundance of reads.

As shown in Figure 3A, diverse cyanobacteria were identified in the lake including genera of filamentous and picocyanobacterial species. The most dominant genera were *Planktothrix* sp.*, Cyanobium and Pseudoanabaena.* Less dominant genera included *Tychonema* and *Microcystis* among other. At the species level, reads belonging to genus *Planktothrix* were identified as *P. rubescens* (Supplementary Figure S5) and those identified as members of genus *Pseudoanabaena* were identified as *Pseudoanabaena foetida*. Reads associated with genus *Cyanobium,* showed higher diversity and were identified as diverse species including *Synechococcus rubescens* and *Cyanobium* sp. (Supplementary Figure S5).

The spatiotemporal abundance of the 4 genera showing highest number of reads in the dataset are shown in Figure 3B. In the top layer of the water column (0–25 meters deep), genus *Planktothrix,* reached a maximum relative abundance of approximately 90% and was dominant in the community until February 2020 (Figures 1A, 3B). The relative abundance of *P. rubescens* remained low from February 2020 (nearly 0%) until October 2020, when it surpassed 25% of all identified bacteria. The spatiotemporal distribution of the genus *Planktothrix* is shown in Figure 3B and suggests the existence of changing ecological niches throughout the year. In autumn and winter, *P. rubescens* was mostly located at 0 to 30 meters depth, while in May and June it became restricted to depths between 15 to 30 meters and was nearly absent from the water column during summer. From March to September 2020, the bacterioplankton communities in the productive layer were dominated by the genera *Flavobacterium*, *CL500-29* and *hgcl clade,* where the cyanobacteria that dominated the column during summertime in the absence of *Planktothrix* was genus *Cyanobium*.

The bottom layer of the water column (from 30 to 100 meters deep) was mainly dominated by the genera *CL500-29*, *Flavobacterium*, *Rhodoferax* and *Methylobacter* throughout the year (Figure 1A). Unlike the top layer, the relative abundance of cyanobacteria was quite low, even in November 2019 when *Planktothrix* bloomed in the bottom layer (maximum relative abundance of 27%). Nevertheless, the relative abundance of *Planktothrix* rapidly increased in December 2019 and January 2020. In March 2020, the bacteria community was suddenly dominated by genus *Flavobacterium* (60%), a proliferation which rapidly decreased by May 2020.

Unlike *P. rubescens*, picocyanobacterial genus *Cyanobium* was mostly restricted to the top of the water column, specifically up to 20 meters of depth and was mostly abundant from July to October (Figure 3B). Conversely, genus *Pseudoanabaena* was typically found at depths from 20 to 40 meters from May to July, although lower abundances were also observed in deeper layers of the water column in winter (February 2020). In general, genus *Aphanizomenon* showed lower relative abundances, but sporadic dominance could be observed from 10 to 30 meters deep in November 2019.

### 3.4 Environmental conditions associated with *Planktothrix rubescens*

**Figure 4:**
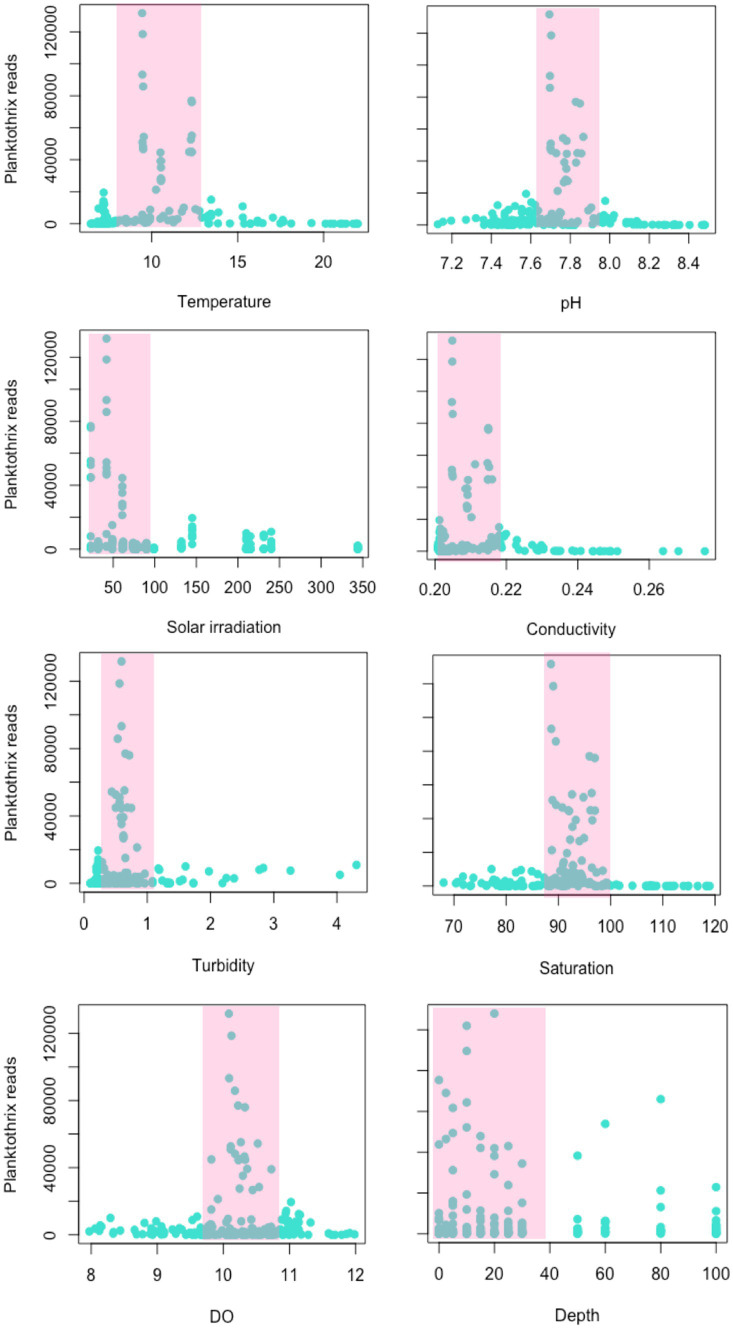
Environmental variables associated with the increase of *Planktothrix rubescens* in the current study. Temperature is presented in Celsius degrees, conductivity is in μS/cm, global solar irradiation in Wm^−2^, turbidity in NTU, oxygen saturation in %, DO in mg/L and depth is in meters. The abundance of *Planktothrix rubescens* reads was extracted for each sample of the study (*n* = 218).

The dominance of *P. rubescens* OTUs was coincident with an observed decrease of water temperature, pH and solar irradiation, and an increase in the wind speed (Supplementary Figure S6). Figure 4 shows the specific environmental values associated with a higher number of reads identified as *P. rubescens*. Specifically, proliferation of *P. rubescens* was associated with low water temperatures (ranging from 9°C–12°C), a pH value of 7.8, low solar irradiation (50 W/m^2^), conductivity values between 0.2–0.22 μS/cm, very low turbidity (0.8 NTU), oxygen saturation between 90%–100%, dissolved oxygen between 10–11 mg/L and mostly associated to the productive layer.

### 3.5 Bacterioplankton biomass and diversity indices

**Figure 5:**
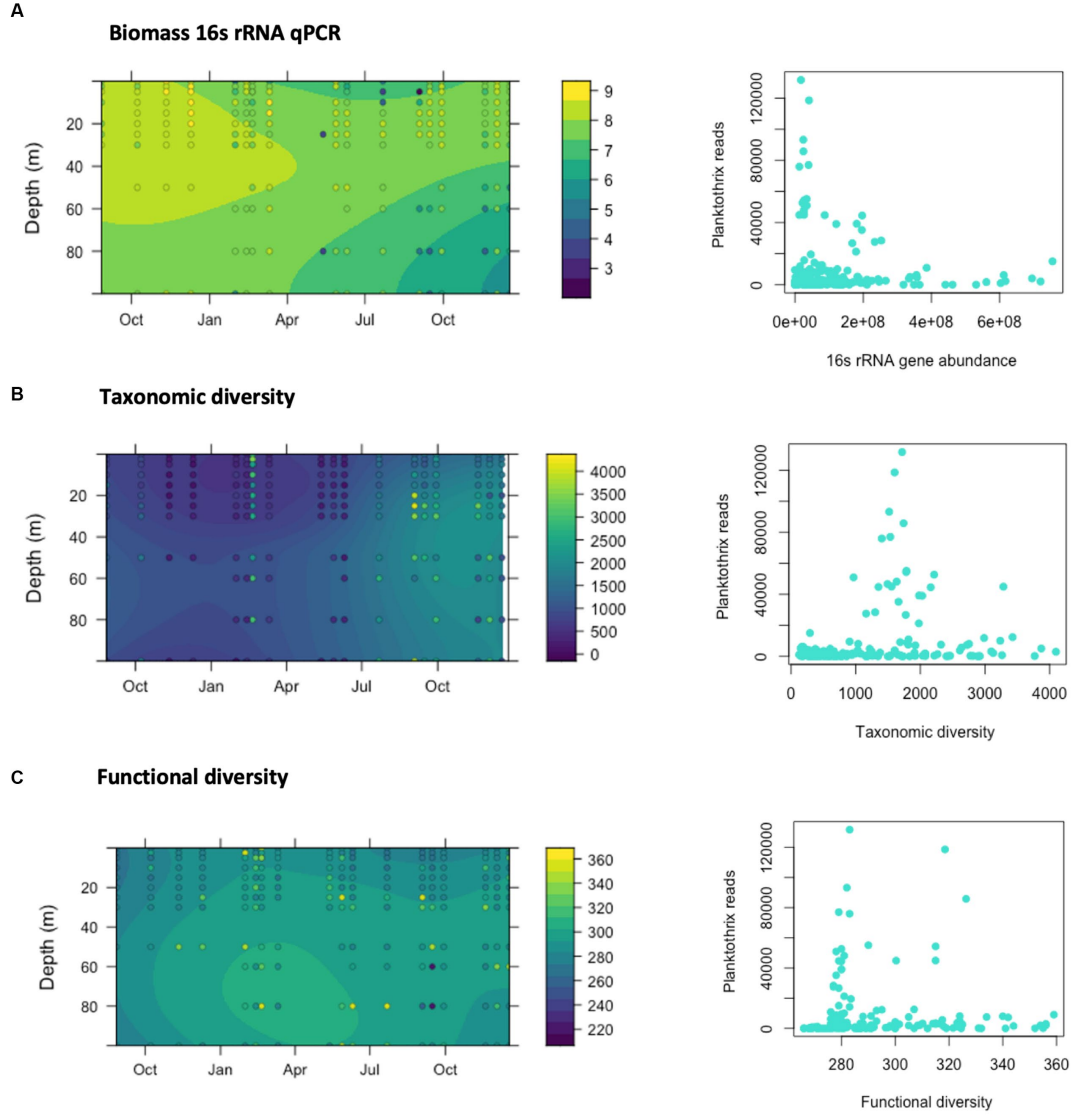
Relationship between the dominance of *Planktothrix rubescens* reads, 16srRNA gene abundance and diversity estimations. **(A)** Abundance of the 16S rRNA gene determined by qPCR in lake samples collected across different depths and dates from August 2019 to December 2020. Results are represented as Log10 genomic copies per liter of lake water. **(B)** Estimates of taxonomic diversity according to Chao1 indices. **(C)** Estimates of functional diversity according to Chao1 indices, calculated from the predicted KEGG pathways obtained using PICRUST2.

We monitored the concentration of 16S rRNA genes using qPCR analysis as a proxy of bacterioplankton biomass abundance (Figure 5A). The concentration of the 16S rRNA gene in the samples ranged from 5.97 × 10^3^ to 5.31 ×10^8^ genome copies (gc)/L and overall, the mean value was 5.31 × 10^7^ gc/L (Figure 5). A peak in the concentration of 16S rRNA gene was observed in January 2020 with values ranging from 2.1 × 10^8^ to nearly 10^9^ gc/L. Bacterioplankton abundance was generally higher in the top of the water column (0–30 m) during periods of stratification (summer, autumn), but equivalent to the values of the bottom layer during deep mixing in winter. Our data shows that higher number of reads identified as *P. rubescens* coincide with the lowest abundances of 16S rRNA gene, and highest estimates of bacteria abundance were only observed when *P. rubescens* reads were very low.

The spatiotemporal distribution of the taxonomic diversity in the studied communities (measured as Chao1 indices) is shown in Figure 5B. Chao1 indices ranged from few hundred up to nearly 4,000 in March, and from July to November 2020. While occasionally higher indices were determined in the bottom layer of the water column, these differences were not statistically significant. Temporal analysis of the taxonomic diversity (Figure 5B) showed higher Chao1 values (up to almost 4,000) during lake stratification in summer and autumn. Lower Chao1 values (below 1,000) were observed from November 2019 until early February 2020 and again from March to June 2020. Taxonomic diversity was positively associated with sunlight, wind speed and phosphorus concentrations, and negatively associated with rain, dissolved oxygen, and chlorophyll a concentration (Supplementary Figure S10).

Functional diversity was also assessed using Chao1 indices and its spatiotemporal distribution along the water column is shown in Figure 5C. Higher functional diversity was observed in the bottom layer, particularly from February to June 2020 and unlike taxonomic diversity, differences observed between top and bottom layers were statistically significant in a *t*-test (*p*-value = 0.0019). The functional diversity of the bacteria communities was higher from January to May 2020, with one exception in March 2020. No clear trend could be identified between functional and taxonomic diversity (Supplementary Figure S9) and functional diversity was positively associated with nutrient concentrations and negatively correlated with temperature, pH, and chlorophyll a concentration (Supplementary Figure S10). As described for the abundance of 16S rRNA gene, high relative abundances of the *P. rubescens* can be associated with lower estimates of PICRUSt2-predicted functional diversity in the water column, and higher diversity was mostly observed at times when the relative abundance of *P. rubescens* was low (Figure 5C).

### 3.6 PICRUSt2-predicted functions associated with the dominance of *Planktothrix rubescens*

In this study, PICRUSt2 predictions were based on 12,748 OTUs and generated a total of 308 features. Low NSTI values (<0.15), suggest that the samples share a close phylogenetic relationship and are well-suited for analysis with PICRUSt2. In our extensive dataset, composed of 218 lake water samples, weighted NSTI values ranged from 0.05 to 0.347 with a mean value of 0.220 ± 0.06, suggesting a reasonable reliability of the obtained predictions (Langille et al., 2013). The most abundant KEGG pathways predicted from the sequencing results belonged to the categories of cellular processes, environmental information processing, genetic information processing and microbial metabolism. Table 1 shows a list of the PICRUSt2-predicted functional pathways that were correlated with the number of reads identified as *P. rubescens*. Overall, *P. rubescens* reads were positively correlated with diverse metabolic functions such as genetic information processing, carbohydrate metabolism, photosynthesis, glycan biosynthesis, amino acid metabolism, degradation of xenobiotics and the biosynthesis of secondary metabolites. Among all these functions, photosynthesis proteins, including antenna proteins, replication, recombination and repair proteins, carbohydrate metabolism, carotenoid biosynthesis and the biosynthesis of macrolides were the ones with higher correlation coefficients.

**Table 1:** PICRUSt2 predicted functional pathways showing significant correlations with *Plankthotrix rubescens* reads.

General processes	Predicted KEGG pathway	Correlation coefficient	*p*-value
Biosynthesis of secondary metabolites	Butirosin and neomycin biosynthesis	0.540521783	4.44E−16
	Isoquinoline alkaloid biosynthesis	0.521254299	7.77E−15
Carbohydrate metabolism	Starch and sucrose metabolism	0.517889545	1.24E−14
Cellular processes and signaling	Sporulation	0.590309636	0
	Signal transduction mechanisms	0.518700664	1.11E−14
Energy metabolism	**Photosynthesis—antenna proteins**	**0.812632671**	0
	**Photosynthesis proteins**	**0.802461181**	0
	**Photosynthesis**	**0.796149456**	0
	Carbon fixation in photosynthetic organisms	0.523303743	5.77E−15
Environmental information processing	Ion channels	0.534312261	1.33E−15
Genetic information processing	**Replication, recombination and repair proteins**	**0.735462236**	0
	Restriction enzyme	0.686880751	0
	RNA transport	0.565224507	0
	RNA degradation	0.514858556	1.87E−14
Glycan biosynthesis and metabolism	N-Glycan biosynthesis	0.612298906	0
	Glycosyltransferases	0.522179583	6.66E−15
Metabolism	**Carbohydrate metabolism**	**0.701319679**	0
	Others	0.637005874	0
	Amino acid metabolism	0.558735588	0
Metabolism of cofactors and vitamins	Ubiquinone and other terpenoid-quinone biosynthesis	0.597394151	0
	Porphyrin and chlorophyll metabolism	0.571232153	0
	Lipoic acid metabolism	0.549014081	0
Metabolism of terpenoids and polyketides	**Carotenoid biosynthesis**	**0.809793148**	0
	**Biosynthesis of 12, 14, and 16-membered macrolides**	**0.749152832**	0
	Biosynthesis of vancomycin group antibiotics	0.526431044	3.77E−15
	Polyketide sugar unit biosynthesis	0.50728973	5.13E−14
Metabolism, enzyme families	Protein kinases	0.659465394	0
	Peptidases	0.512782765	2.46E−14
Xenobiotics degradation and metabolism	Chlorocyclohexane and chlorobenzene degradation	0.65212298	0
	Atrazine degradation	0.555279076	0

All *p*-values were <4.44e10^–14^ and only correlations with Pearson correlation coefficient larger than 0.50 and pertinent to prokaryotes are shown here. KEGG pathways with coefficients larger than 0.7 are highlighted in bold.

**Figure 6:**
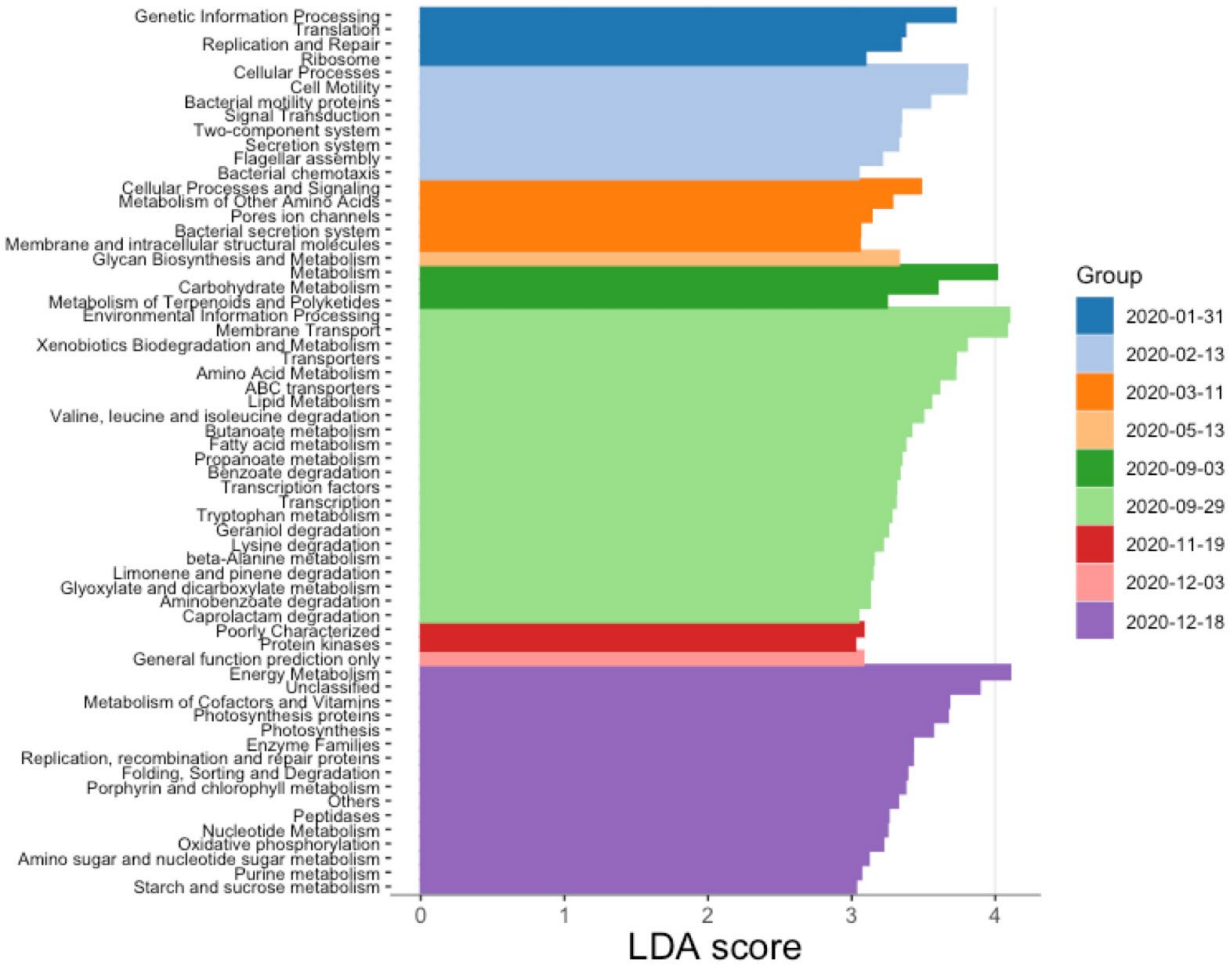
PICRUSt-predicted functional markers for different dates. The determination of the markers was done using Lefse algorithm based on KEGG pathway predictions obtained with PICRUSt2. LDA stands for linear discriminant analysis.

Differential abundance tests implemented in LefSe allowed us to identify taxonomic groups and functional pathways that characterized diverse sampling dates (LDA scores above 3; Supplementary Figure S8; Figure 6). These analyses show that in January 2020, 2 months after the start of the dominance of *P. rubescens*, functional markers in the water column were related to the processing of genetic information, including functions involved in replication and repair, as well as translation. From February 2020, the water column became enriched in functions relating to chemotaxis, cell motility and secretion. March 2020 was characterized by an enrichment in cellular processes and signaling, metabolism of amino acids, bacterial secretion and the synthesis of membrane and intracellular molecules. No functional biomarkers were identified for months between May and September 2020. In September 2020, the water column had shifted to display a wide diversity of metabolic pathways such as those involved in the degradation of xenobiotics, amino acids, lipids, and the synthesis of transporters. Later in December 2020, the water column showed a new increase in the abundance of functions related to photosynthesis, genetic information processing and repair, as well as the metabolism of amino acids, sugars, and other cofactors.

## 4 Discussion

In this study we characterized the diversity and spatiotemporal dynamics of cyanobacteria communities in Lake Geneva, a large deep mesotrophic lake, and explored the environmental and biological factors that may be associated with dominance of *P. rubescens* in the water column. We confirmed that *P. rubescens* is the dominant cyanobacteria in the lake and that its blooms reduce bacterioplankton abundance and diversity leading to community divergence. Our results showed that the top layer of the water column (spanning from 0 to 30 meters depth) was particularly enriched with three genera of cyanobacteria (*Planktothrix*, *Cyanobium* and *Pseudoanabaena*), as well as *Limnohabitans* (a member of phylum *Verrucomicrobia*) (Kraemer et al., 2021). The most abundant bacteria genera were *CL500-29 marine group*, *Flavobacterium*, *Planktothrix*, *CL500-3*, *hgcl clade* and *Rhodoferax,* which are consistent with genera typically found in other freshwater ecosystems (Newton et al., 2011). Reads belonging to genus *Planktothrix* were exclusively identified as *P. rubescens*, confirming the widespread and dominance of this potentially toxic cyanobacteria in Lake Geneva, as previously described in Lake Zurich and other lakes recovering from eutrophication in the area (Jacquet et al., 2005; Fernández Castro et al., 2021). Our results also highlighted the previously unknown dominance of diverse picocyanobacterial species in the lake belonging to genus *Cyanobium*, which appear particularly abundant in the top of the water column during stratification periods. Reads classified as members of genus *Pseudoanabaena* could be identified as *Pseudoanabaena foetida* and were mostly abundant during May and June at depths from 10 to 30 meters.

As expected from these sequencing observations, a high abundance of PICRUSt2-predicted functional pathways dedicated to photosynthesis and the metabolism of cofactors and vitamins was identified in top layers of the water column, where light conditions are favorable for the proliferation of cyanobacteria. While cyanobacteria were dominant in shallow water layers, the bottom layer (from 30 to 100 meters deep) was instead characterized by a high relative abundance of non-photosynthetic bacteria such as genera *Nitrospira* (important in nitrification) and *Methylobacter* (motile bacteria involved in methane metabolism) (Tsagkari and Sloan, 2018; van Grinsven et al., 2020; Bayer et al., 2021). At the functional level, bacteria in this layer appeared particularly active in cellular processes including cell motility, membrane transport and the metabolism of carbohydrates, which may be released from the lake’s sediments due to the degradation of organic matter and during the sedimentation process (She et al., 2021). Previous research in Lake Baikal (Russia) Soutourina et al. (2001) has shown that warm temperatures may inhibit the motility of bacteria isolates, thus it possible that bacteria motility in Lake Geneva’s bottom layers is favored by cold and stable temperatures.

The study revealed seasonal variations in the abundance of different genera. *Planktothrix* dominated during late autumn and winter, followed by an increase in *Pseudanabaena* towards the end of winter. In spring, there was a notable increase in the relative abundance of *Cyanobium*, especially in the upper water layers, which continued to dominate during the summer across different depths. Previous research conducted investigating the competitive dynamics between *Planktothrix* and *Pseudoanabaena* provided insights indicating that temperature alone does not emerge as the primary determinant influencing the displacement one genus by the other (Su et al., 2022). Instead, this observation suggests that the ecological succession may, in part, be elucidated by the differential light requirements of these two genera. Notably, previous monitoring campaigns conducted by the International Commission for the protection of the waters of Lake Geneva (CIPEL) have demonstrated a late-winter augmentation in phytoplankton abundance in the lake (CIPEL, 2020). The biomass of phytoplankton in water bodies impacts the light extinction coefficient, thereby influencing the distribution of short-wave radiation (Rinke et al., 2010). Although both genera were identified as specialists adapted to low-irradiance conditions, *Pseudanabaena* may exhibit lower light requirements compared to *Planktothrix*.

In stratified lakes, nutrient availability may be diminished in the epilimnion due to their confinement in deeper layers (Zhang et al., 2021). The observed rise in the relative prevalence of genus *Cyanobium* during late spring could be attributed to the scarcity of nutrients within the upper regions of the water column. The small size of picocyanobacteria, combined with their relatively expansive surface area, endows them with exceptional efficiency and resilience, potentially accounting for their widespread distribution and prevalence (Callieri and Stockner, 2000). Their cell size, in conjunction with minimal diffusion boundary layers and a heightened surface area per unit volume, confers upon these minuscule phytoplankton cells a competitive advantage in nutrient poor waters, facilitating enhanced nutrient absorption and efficient utilization for growth and maintenance (Davey et al., 2008). Moreover, owing to their prokaryotic cellular structure, they demand less energy for metabolic upkeep (Quiroga et al., 2021). To provide a comparative perspective, research by Padisák et al. (2003) underscores that the surface-to-volume ratio of *Planktothrix* stands at 0.57, whereas that of *Cyanobium* is substantially higher at 6.73 (Padisák et al., 2003). This disparity might elucidate the inverse correlation observed between *Cyanobium* and nutrient levels, implying that nutrient availability may not act as a limiting factor for this genus, in contrast to its potential impact on *Pseudanabaena* and *Planktothrix*.

Beta-diversity analyses revealed that an increase of community dissimilarity was coincident with sampling dates in which genera *Planktothrix* and *Flavobacterium* were dominant in number of reads. *Planktothrix* is a potentially toxic cyanobacteria known in temperate freshwater ecosystems for its capacity of producing harmful blooms and cyanotoxins, such as microcystins, that cause significant ecological and human health impacts (Blom et al., 2003; Knapp et al., 2021). In this study, dominance (understood as high relative abundance of reads) of *P. rubescens* began in November 2019, associated with a decrease in water temperature, pH and solar irradiation, and an increase in wind speed, which may have contributed to the dispersion of *Planktothrix* filaments from a metalimnetic refuge layer to the rest of the water column, as described for Lake Zurich (Fernández Castro et al., 2021). In this study, the proliferation of *Planktothrix* was associated with very specific environmental conditions: water temperatures ranging from 9°C to 12°C, pH of 7.8, solar irradiation of around 50 W/m^2^, conductivity values between 0.2–0.22 μS/cm, very low turbidity (0.8 NTU), oxygen saturation between 90%–100% and dissolved oxygen values between 10–11 mg/L. In consistence, other authors have also identified the role of solar irradiance and light winds as drivers for the seasonal cycling of *P. rubescens* in Lake Zurich (Fernández Castro et al., 2021; Knapp et al., 2021).

Our results showed that the proliferation of *Planktothrix* was associated with lower 16S rRNA gene abundances, and lower taxonomic and functional diversity estimates as determined by Chao1 indices. Notably, both taxonomic and functional diversity recovered to achieve their maximum annual values upon resolution of the *Planktothrix* bloom. This may suggest that the effects of *Planktothrix* proliferation on bacterioplankton diversity may be only observed over short time periods, and that blooms could ultimately promote functional diversity in the community. This is a novel hypothesis that requires further research. In agreement with the observations of this study, cyanobacteria blooms in lakes have been previously known to have a significant impact on the diversity and functions of bacteria communities. For instance, a study conducted on Lake Agawam (United States) found that cyanobacteria and bacteria abundances have an inverse relationship (Jankowiak and Gobler, 2020). Similarly, recent studies have linked the dynamics of certain cyanobacterial species with microbial community successions, changes in diversity, and dynamic functions during harmful cyanobacterial blooms in a freshwater lake and in lake sediments (Woodhouse et al., 2016; Li et al., 2020; Yang et al., 2021).

The effects of cyanobacteria, and more specifically *Planktothrix*, on specific bacterioplankton functions have previously received less attention in the literature. Here, proliferation of *P. rubescens* was associated with higher abundance PICRUSt2-predicted pathways involved in photosynthesis, replication and repair pathways, metabolism of carbohydrates and the biosynthesis of carotenoid and macrolides [compounds that are share similarities of structure, assembly, and chemical composition with microcystins (Tillett et al., 2000; Barboza et al., 2017; Bouaïcha et al., 2019)]. These functions are consistent with the function of *Planktothrix* as a photosynthetic bacterium, which may need to replicate efficiently to produce blooms and repair its genome from the harmful effects of sunlight, thus producing microcystins and accumulating carbohydrates for modulating buoyancy in the water column (Utkilen et al., 1985). LefSe analyses showed that the proliferation of *Planktothrix* induced seasonal changes on the functional profile of the studied water column. For example, in winter we found an increase in PICRUSt2-predicted functions involving genetic information processing, cell motility and chemotaxis, as well as photosynthesis and sugar metabolism. Conversely, in summer, the bacteria communities in the water column were associated with the metabolism of certain compounds such as xenobiotics, amino acids and lipids. During its autumn proliferation, *Planktothrix* was mostly found in the top 30 meters, progressing down the water column through winter deep mixing. During spring and summer, *Planktothrix* likely regulated its vertical position in the water column using gas vesicles and other physiological changes (Utkilen et al., 1985) to find refuge at deeper layers (around 20–30 meters), close to the metalimnion with suitable irradiation conditions (Knapp et al., 2021).

Our sequencing results suggest an increase of the dominance of genus Flavobacterium after the bloom of *P. rubescens*. Increase of *Flavobacterium* reads was associated with higher pH conditions, abundant precipitations, low water temperatures and low solar irradiation. *Flavobacteria* (family Flavobacteriaceae, phylum Bacteroidota), are non-spore-forming, strictly aerobic, motile by gliding and can degrade complex organic compounds such as polysaccharides (Fernández-Gómez et al., 2013; Waśkiewicz and Irzykowska, 2014; Kraut-Cohen et al., 2021). *Flavobacterium* have been previously described as main responders to phytoplankton blooms and were the dominant heterotrophic bacteria in the cyanobacteria phycosphere during blooms (Kim et al., 2019, 2020; Bartlau et al., 2022). We hypothesize that the proliferation of genus *Flavobacterium* observed in this study occurred as a response to the bloom of *P. rubescens* and an increase of water temperature and sunlight irradiation, and that they contributed to the degradation of dead *P. rubescens* filaments. The increase of *Flavobacterium* was associated with PICRUSt2-predicted functional pathways involved in the metabolism of amino acids, secretion systems and the synthesis of membrane and intracellular structural molecules, which may serve *Flavobacterium* to degrade organic matter in the water column.

To conclude, our study has shed light on the diversity and dynamics of cyanobacteria communities in Lake Geneva and confirmed the dominance of *P. rubescens* in this mesotrophic lake. In addition, our study has also shown that picocyanobacteria may be more relevant for the lake’s ecology than previously thought. Overall, these observations emphasize the need for further cyanobacteria monitoring in Lake Geneva, which serves a source of drinking water for approximately 900,000 people (Huisman et al., 2018). Particularly, with regards to the production of cyanotoxins by the herein identified species. Moreover, we have identified environmental conditions that may favor the proliferation of *P. rubescens* and established a link between dominance of *P. rubescens* and community dissimilarity, and the bacterioplankton biomass and diversity. These observations have some implications for the biological consequences of climate change. Global warming is expected to prolong lake stratification periods, which may induce abnormal oxygenation and nutrient dynamics within the water column (Weckström et al., 2016). Our findings suggest that summer stratification and winter deep mixing are important events that influence the proliferation of *P. rubescens*, as well as the abundance, composition, diversity and functioning of a deep mesotrophic lake such as Lake Geneva. In Lake Geneva, the proliferation of *P. rubescens* led to a loss of bacteria diversity along the entire water column in the short-term. While we were able to show that the functional richness of the community was maintained, and even stimulated by the bloom of *P. rubescens*, it is still necessary to better understand the environmental drivers and ecological consequences of these blooms to better anticipate the potential biological consequences of climate change in non-eutrophic freshwater ecosystems.

## Data availability statement

Raw sequence data are available at NCBI with a BioProject ID PRJNA974682 and accession numbers from SAMN36273484 to SAMN36273698. The obtained OTU table, taxon identification table and environmental data on the water column are deposited in Zenodo repository with number (doi: 10.5281/zenodo.7957172).

## Author contributions

AC: Conceptualization, Funding acquisition, Investigation, Supervision, Writing – original draft, Writing – review & editing. CC: Methodology, Writing – review & editing. OS: Formal analysis, Writing – review & editing. AG: Data curation, Writing – review & editing. HC: Methodology, Writing – review & editing. NP: Methodology, Writing – review & editing. CW: Data curation, Writing – review & editing. TK: Formal analysis, Resources, Writing – review & editing. SJ: Writing – review & editing.

## References

[ref1] Amorim C. A. Moura A. N. (2021). Ecological impacts of freshwater algal blooms on water quality, plankton biodiversity, structure, and ecosystem functioning. Sci. Total Environ. 758:143605. doi: 10.1016/j.scitotenv.2020.143605, PMID: 33248793

[ref2] Barboza G. Gorlach-Lira K. Sassi C. Sassi R. (2017). Microcystins production and antibacterial activity of cyanobacterial strains of *Synechocystis*, *Synechococcus* and *Romeria* isolated from water and coral reef organisms of Brazilian coast. Rev. Biol. Trop. 65:890. doi: 10.15517/rbt.v65i3.29437

[ref3] Bartlau N. Wichels A. Krohne G. Adriaenssens E. M. Heins A. Fuchs B. M.. (2022). Highly diverse flavobacterial phages isolated from North Sea spring blooms. ISME J. 16, 555–568. doi: 10.1038/s41396-021-01097-4, PMID: 34475519 PMC8776804

[ref4] Bayer B. Saito M. A. McIlvin M. R. Lücker S. Moran D. M. Lankiewicz T. S.. (2021). Metabolic versatility of the nitrite-oxidizing bacterium *Nitrospira marina* and its proteomic response to oxygen-limited conditions. ISME J. 15, 1025–1039. doi: 10.1038/s41396-020-00828-3, PMID: 33230266 PMC8115632

[ref5] Berdjeb L. Ghiglione J. F. Jacquet S. (2011). Bottom-up versus top-down control of hypo- and epilimnion free-living bacterial community structures in two neighboring freshwater lakes. Appl. Environ. Microbiol. 77, 3591–3599. doi: 10.1128/AEM.02739-10, PMID: 21478309 PMC3127590

[ref6] Blom J. F. Bister B. Bischoff D. Nicholson G. Jung G. Süssmuth R. D.. (2003). Oscillapeptin J, a new grazer toxin of the freshwater cyanobacterium *Planktothrix rubescens*. J. Nat. Prod. 66, 431–434. doi: 10.1021/np020397f, PMID: 12662108

[ref7] Bolyen E. Rideout J. R. Dillon M. R. Bokulich N. A. Abnet C. C. Al-Ghalith G. A.. (2019). Reproducible, interactive, scalable and extensible microbiome data science using QIIME 2. Nat. Biotechnol. 37, 852–857. doi: 10.1038/s41587-019-0209-9 31341288 PMC7015180

[ref8] Bouaïcha N. Miles C. Beach D. Labidi Z. Djabri A. Benayache N.. (2019). Structural diversity, characterization and toxicology of microcystins. Toxins 11:714. doi: 10.3390/toxins11120714, PMID: 31817927 PMC6950048

[ref9] Callieri C. Stockner J. (2000). Picocyanobacteria success in oligotrophic lakes: fact or fiction? J. Limnol. 59:72. doi: 10.4081/jlimnol.2000.72

[ref10] Chorus I. Welker M. Toxic cyanobacteria in water (2021) London CRC Press

[ref11] CIPEL. Rapport-scientifique CIPEL 2020–2021 (2020).

[ref12] Darrin S. Bryan C. (2021). “Data visualization with ggplot” in Probability, statistics, and data

[ref13] Davey M. Tarran G. A. Mills M. M. Ridame C. Geider R. J. LaRoche J. (2008). Nutrient limitation of picophytoplankton photosynthesis and growth in the tropical North Atlantic. Limnol. Oceanogr. 53, 1722–1733. doi: 10.4319/lo.2008.53.5.1722

[ref14] Douglas G. M. Maffei V. J. Zaneveld J. R. Yurgel S. N. Brown J. R. Taylor C. M.. (2020). PICRUSt2 for prediction of metagenome functions. Nat. Biotechnol. 38, 685–688. doi: 10.1038/s41587-020-0548-6, PMID: 32483366 PMC7365738

[ref15] Ernst B. Hoeger S. J. O’Brien E. Dietrich D. R. (2009). Abundance and toxicity of *Planktothrix rubescens* in the pre-alpine Lake Ammersee, Germany. Harmful Algae 8, 329–342. doi: 10.1016/j.hal.2008.07.006

[ref16] Ezzedine J. A. Jacas L. Desdevises Y. Jacquet S. (2020). *Bdellovibrio* and like organisms in Lake Geneva: an unseen elephant in the room? Front. Microbiol. 11:98. doi: 10.3389/fmicb.2020.00098, PMID: 32117128 PMC7034301

[ref17] Fenchel T. (2002). Microbial behavior in a heterogeneous world. Science 296, 1068–1071. doi: 10.1126/science.1070118, PMID: 12004118

[ref18] Fernández Castro B. Sepúlveda Steiner O. Knapp D. Posch T. Bouffard D. Wüest A. (2021). Inhibited vertical mixing and seasonal persistence of a thin cyanobacterial layer in a stratified lake. Aquat. Sci. 83, 351–365. doi: 10.1007/s00027-021-00785-9

[ref19] Fernández-Gómez B. Richter M. Schüler M. Pinhassi J. Acinas S. G. González J. M.. (2013). Ecology of marine bacteroidetes: a comparative genomics approach. ISME J. 7, 1026–1037. doi: 10.1038/ismej.2012.169, PMID: 23303374 PMC3635232

[ref20] Fox J. Weisberg S. An R companion to applied regression, 3. Thousand Oaks, CA: Sage. (2019).

[ref21] Gallina N. Beniston M. Jacquet S. (2017). Estimating future cyanobacterial occurrence and importance in lakes: a case study with *Planktothrix rubescens* in Lake Geneva. Aquat. Sci. 79, 249–263. doi: 10.1007/s00027-016-0494-z

[ref22] Grothendieck G. (2008). Lattice: multivariate data visualization with R. J. Stat. Softw. 25, 1–3. doi: 10.18637/jss.v025.b02

[ref23] Guedes I. A. Rachid C. T. C. C. Rangel L. M. Silva L. H. S. Bisch P. M. Azevedo S. M. F. O.. (2018). Close link between harmful cyanobacterial dominance and associated bacterioplankton in a tropical eutrophic reservoir. Front. Microbiol. 9:424. doi: 10.3389/fmicb.2018.00424, PMID: 29593677 PMC5857610

[ref24] Hijmans R. J. van Etten J. raster: geographic analysis and modeling with raster data. R package version 2.5-2 (2015).

[ref25] Huisman J. Codd G. A. Paerl H. W. Ibelings B. W. Verspagen J. M. H. Visser P. M. (2018). Cyanobacterial blooms. Nat. Rev. Microbiol. 16, 471–483. doi: 10.1038/s41579-018-0040-1 29946124

[ref26] Jacquet S. Briand J. F. Leboulanger C. Avois-Jacquet C. Oberhaus L. Tassin B.. (2005). The proliferation of the toxic cyanobacterium *Planktothrix rubescens* following restoration of the largest natural French lake (Lac du Bourget). Harmful Algae 4, 651–672. doi: 10.1016/j.hal.2003.12.006

[ref27] James G. Witten D. Hastie T. Introduction to statistical learning with applications in R. Synthesis lectures on mathematics and statistics volume 11 (2019).

[ref28] Jankowiak J. G. Gobler C. J. (2020). The composition and function of microbiomes within *Microcystis* colonies are significantly different than native bacterial assemblages in two north American Lakes. Front. Microbiol. 11:1016. doi: 10.3389/fmicb.2020.01016, PMID: 32547511 PMC7270213

[ref29] Jia J. Shi W. Chen Q. Lauridsen T. L. (2017). Spatial and temporal variations reveal the response of zooplankton to cyanobacteria. Harmful Algae 64, 63–73. doi: 10.1016/j.hal.2017.02.008, PMID: 28427573

[ref30] Kanehisa M. Goto S. K. E. G. G. (2000). Kyoto encyclopedia of genes and genomes. Nucleic Acids Res. 28, 27–30. doi: 10.1093/nar/28.1.27, PMID: 10592173 PMC102409

[ref31] Kim M. Lee J. Yang D. Park H. Y. Park W. (2020). Seasonal dynamics of the bacterial communities associated with cyanobacterial blooms in the Han River. Environ. Pollut. 266:115198. doi: 10.1016/j.envpol.2020.115198, PMID: 32668373

[ref32] Kim M. Shin B. Lee J. Park H. Y. Park W. (2019). Culture-independent and culture-dependent analyses of the bacterial community in the phycosphere of cyanobloom-forming *Microcystis aeruginosa*. Sci. Rep. 9:20416. doi: 10.1038/s41598-019-56882-1, PMID: 31892695 PMC6938486

[ref33] Klindworth A. Pruesse E. Schweer T. Peplies J. Quast C. Horn M.. (2013). Evaluation of general 16S ribosomal RNA gene PCR primers for classical and next-generation sequencing-based diversity studies. Nucleic Acids Res. 41:e1. doi: 10.1093/nar/gks808, PMID: 22933715 PMC3592464

[ref34] Knapp D. Fernández Castro B. Marty D. Loher E. Köster O. Wüest A.. (2021). The red harmful plague in times of climate change: blooms of the cyanobacterium *Planktothrix rubescens* triggered by stratification dynamics and irradiance. Front. Microbiol. 12:705914. doi: 10.3389/fmicb.2021.705914, PMID: 34512582 PMC8425285

[ref35] Kraemer B. M. Pilla R. M. Woolway R. I. Anneville O. Ban S. Colom-Montero W.. (2021). Climate change drives widespread shifts in lake thermal habitat. Nat. Clim. Chang. 11, 521–529. doi: 10.1038/s41558-021-01060-3

[ref36] Kraut-Cohen J. Shapiro O. H. Dror B. Cytryn E. (2021). Pectin induced colony expansion of soil-derived *Flavobacterium* strains. Front. Microbiol. 12:065981. doi: 10.3389/fmicb.2021.651891, PMID: 33889143 PMC8056085

[ref37] Krztoń W. Kosiba J. Pociecha A. Wilk-Woźniak E. (2019). The effect of cyanobacterial blooms on bio- and functional diversity of zooplankton communities. Biodivers. Conserv. 28, 1815–1835. doi: 10.1007/s10531-019-01758-z

[ref38] Langille M. G. I. Zaneveld J. Caporaso J. G. McDonald D. Knights D. Reyes J. A.. (2013). Predictive functional profiling of microbial communities using 16S rRNA marker gene sequences. Nat. Biotechnol. 31, 814–821. doi: 10.1038/nbt.2676, PMID: 23975157 PMC3819121

[ref39] Li H. Barber M. Lu J. Goel R. (2020). Microbial community successions and their dynamic functions during harmful cyanobacterial blooms in a freshwater lake. Water Res. 185:116292. doi: 10.1016/j.watres.2020.116292, PMID: 33086464 PMC7737503

[ref40] Liu C. Cui Y. Li X. Yao M. (2021). Microeco: an R package for data mining in microbial community ecology. FEMS Microbiol. Ecol. 97:fiaa255. doi: 10.1093/femsec/fiaa255, PMID: 33332530

[ref41] Mankiewicz-Boczek J. Font-Nájera A. (2022). Temporal and functional interrelationships between bacterioplankton communities and the development of a toxigenic *Microcystis* bloom in a lowland European reservoir. Sci. Rep. 12:19332. doi: 10.1038/s41598-022-23671-2, PMID: 36369518 PMC9652341

[ref42] Minaudo C. Odermatt D. Bouffard D. Rahaghi A. I. Lavanchy S. Wüest A. (2021). The imprint of primary production on high-frequency profiles of Lake optical properties. Environ. Sci. Technol. 55, 14234–14244. doi: 10.1021/acs.est.1c02585, PMID: 34591466

[ref43] Newton R. J. Jones S. E. Eiler A. McMahon K. Bertilsson S. (2011). A guide to the natural history of freshwater lake bacteria. Microbiol. Mol. Biol. Rev. 75, 14–49. doi: 10.1128/MMBR.00028-10, PMID: 21372319 PMC3063352

[ref44] Padisák J. Barbosa F. Koschel R. Krienitz L. (2003). Deep layer cyanoprokaryota maxima in temperate and tropical lakes. Adv. Limnol. 58, 175–199.

[ref45] Paerl H. W. Otten T. G. (2013). Harmful cyanobacterial blooms: causes, consequences, and controls. Microb. Ecol. 65, 995–1010. doi: 10.1007/s00248-012-0159-y, PMID: 23314096

[ref46] Paerl H. W. Paul V. J. (2012). Climate change: links to global expansion of harmful cyanobacteria. Water Res. 46, 1349–1363. doi: 10.1016/j.watres.2011.08.002, PMID: 21893330

[ref47] Parvathi A. Zhong X. Ram A. S. P. Jacquet S. Jacquet S. (2014). Dynamics of auto-and heterotrophic picoplankton and associated viruses in Lake Geneva. Hydrol. Earth Syst. Sci. 18, 1073–1087. doi: 10.5194/hess-18-1073-2014

[ref48] Posch T. Köster O. Salcher M. M. Pernthaler J. (2012). Harmful filamentous cyanobacteria favoured by reduced water turnover with lake warming. Nat. Clim. Chang. 2, 809–813. doi: 10.1038/nclimate1581

[ref49] Quiroga M. V. Huber P. Ospina-Serna J. Diovisalvi N. Odriozola M. Cueto G. R.. (2021). The dynamics of picocyanobacteria from a hypereutrophic shallow lake is affected by light-climate and small-bodied zooplankton: a 10 years cytometric time-series analysis. FEMS Microbiol. Ecol. 97:fiab055. doi: 10.1093/femsec/fiab055, PMID: 33784379

[ref50] R Core Team. R: A language and environment for statistical computing (R foundation for statistical computing, Vienna, (2012). Available at: http://www.R-project.org (2015).

[ref51] Rinke K. Yeates P. Rothhaupt K. O. (2010). A simulation study of the feedback of phytoplankton on thermal structure via light extinction. Freshw. Biol. 55, 1674–1693. doi: 10.1111/j.1365-2427.2010.02401.x

[ref52] Segata N. Izard J. Waldron L. Gevers D. Miropolsky L. Garrett W. S.. (2011). Metagenomic biomarker discovery and explanation. Genome Biol. 12:R60. doi: 10.1186/gb-2011-12-6-r60, PMID: 21702898 PMC3218848

[ref53] She Z. Wang J. He C. Pan X. Li Y. Zhang S.. (2021). The stratified distribution of dissolved organic matter in an AMD lake revealed by multi-sample evaluation procedure. Environ. Sci. Technol. 55, 8401–8409. doi: 10.1021/acs.est.0c05319 34060313

[ref54] Soutourina O. A. Semenova E. A. Parfenova V. Danchin A. Bertin P. (2001). Control of bacterial motility by environmental factors in polarly flagellated and peritrichous bacteria isolated from Lake Baikal. Appl. Environ. Microbiol. 67, 3852–3859. doi: 10.1128/AEM.67.9.3852-3859.2001, PMID: 11525977 PMC93101

[ref55] Su M. Zhu Y. Andersen T. Wang X. Yu Z. Lu J.. (2022). Light-dominated selection shaping filamentous cyanobacterial assemblages drives odor problem in a drinking water reservoir. npj Clean Water 5:37. doi: 10.1038/s41545-022-00181-2

[ref56] Sukenik A. Quesada A. Salmaso N. (2015). Global expansion of toxic and non-toxic cyanobacteria: effect on ecosystem functioning. Biodivers. Conserv. 24, 889–908. doi: 10.1007/s10531-015-0905-9

[ref57] Tillett D. Dittmann E. Erhard M. von Döhren H. Börner T. Neilan B. A. (2000). Structural organization of microcystin biosynthesis in *Microcystis aeruginosa* PCC7806: an integrated peptide-polyketide synthetase system. Chem. Biol. 7, 753–764. doi: 10.1016/S1074-5521(00)00021-1, PMID: 11033079

[ref58] Tsagkari E. Sloan W. T. (2018). The role of the motility of methylobacterium in bacterial interactions in drinking water. Water 10:1386. doi: 10.3390/w10101386

[ref59] Utkilen H. C. Oliver R. L. Walsby A. E. (1985). Buoyancy regulation in a red Oscillatoria unable to collapse gas vacuoles by turgor pressure. Arch. Hydrobiol. 102, 319–329. doi: 10.1127/archiv-hydrobiol/102/1985/319

[ref60] van Grinsven S. Sinninghe Damsté J. S. Abdala Asbun A. Engelmann J. C. Harrison J. Villanueva L. (2020). Methane oxidation in anoxic lake water stimulated by nitrate and sulfate addition. Environ. Microbiol. 22, 766–782. doi: 10.1111/1462-2920.14886 31814267 PMC7027835

[ref61] Walsby A. E. (2005). Stratification by cyanobacteria in lakes: a dynamic buoyancy model indicates size limitations met by *Planktothrix rubescens* filaments. New Phytol. 168, 365–376. doi: 10.1111/j.1469-8137.2005.01508.x, PMID: 16219076

[ref62] Walsby A. E. Ng G. Dunn C. Davis P. A. (2004). Comparison of the depth where *Planktothrix rubescens* stratifies and the depth where the daily insolation supports its neutral buoyancy. New Phytol. 162, 133–145. doi: 10.1111/j.1469-8137.2004.01020.x

[ref63] Walsby A. E. Schanz F. Schmid M. (2006). The Burgundy-blood phenomenon: a model of buoyancy change explains autumnal waterblooms by *Planktothrix rubescens* in Lake Zürich. New Phytol. 169, 109–122. doi: 10.1111/j.1469-8137.2005.01567.x, PMID: 16390423

[ref64] Waśkiewicz A. Irzykowska L. (2014). “*Flavobacterium* spp.—characteristics, occurrence, and toxicity” in Encyclopedia of food microbiology. 2nd ed

[ref65] Weckström K. Weckström J. Huber K. Kamenik C. Schmidt R. Salvenmoser W.. (2016). Impacts of climate warming on alpine lake biota over the past decade. Arct. Antarct. Alp. Res. 48, 361–376. doi: 10.1657/AAAR0015-058

[ref66] Woodhouse J. N. Kinsela A. S. Collins R. N. Bowling L. C. Honeyman G. L. Holliday J. K.. (2016). Microbial communities reflect temporal changes in cyanobacterial composition in a shallow ephemeral freshwater lake. ISME J. 10, 1337–1351. doi: 10.1038/ismej.2015.218, PMID: 26636552 PMC5029192

[ref67] Wüest A. Bouffard D. Guillard J. Ibelings B. W. Lavanchy S. Perga M. E.. (2021a). LéXPLORE: a floating laboratory on Lake Geneva offering unique lake research opportunities. Wiley Interdiscip. Rev. Water 8:e1544. doi: 10.1002/wat2.1544

[ref68] Wüest A. Bouffard D. Guillard J. Ibelings B. W. Lavanchy S. Perga M.-E.. (2021b). A floating laboratory on Lake Geneva offering unique lake research opportunities. Wiley Interdiscip. Rev. Water 8:2022:e1572. doi: 10.1002/wat2.1572, (Erratum)

[ref69] Yang Y. Chen J. Chen X. Jiang Q. Liu Y. Xie S. (2021). Cyanobacterial bloom induces structural and functional succession of microbial communities in eutrophic lake sediments. Environ. Pollut. 284:117157. doi: 10.1016/j.envpol.2021.117157 33892464

[ref70] Yankova Y. Villiger J. Pernthaler J. Schanz F. Posch T. (2016). Prolongation, deepening and warming of the metalimnion change habitat conditions of the harmful filamentous cyanobacterium *Planktothrix rubescens* in a prealpine lake. Hydrobiologia 776, 125–138. doi: 10.1007/s10750-016-2745-3

[ref71] Zhang J. Kong J. D. Shi J. Wang H. (2021). Phytoplankton competition for nutrients and light in a stratified lake: a mathematical model connecting epilimnion and hypolimnion. J. Nonlinear Sci. 31:35. doi: 10.1007/s00332-021-09693-6

[ref72] Zheng X. Xiao L. Ren J. Yang L. (2008). The effect of a *Microcystis aeruginosa* bloom on the bacterioplankton community composition of lake Xuanwa. J. Freshw. Ecol. 23, 297–304. doi: 10.1080/02705060.2008.9664202

