# Supplementary material for: Vertical distribution and seasonal dynamics of planktonic cyanobacteria communities in a water column of deep mesotrophic Lake Geneva
Source: Front Microbiol. 2023 Dec 15;14:1295193. doi: 10.3389/fmicb.2023.1295193 (PMC10758419; doi:10.3389/fmicb.2023.1295193)

Fig. S1. Lake Geneva and LéXPLORE scientific platform located in Pully, Switzerland. (A) Photo of the platform taken on 26th January 2021. (B) Location of our sampling site (red marker) in Lake Geneva. Map created with QGIS version 3.18.

A

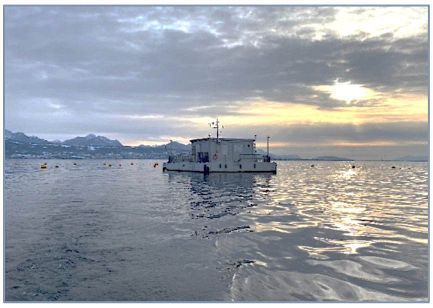

B

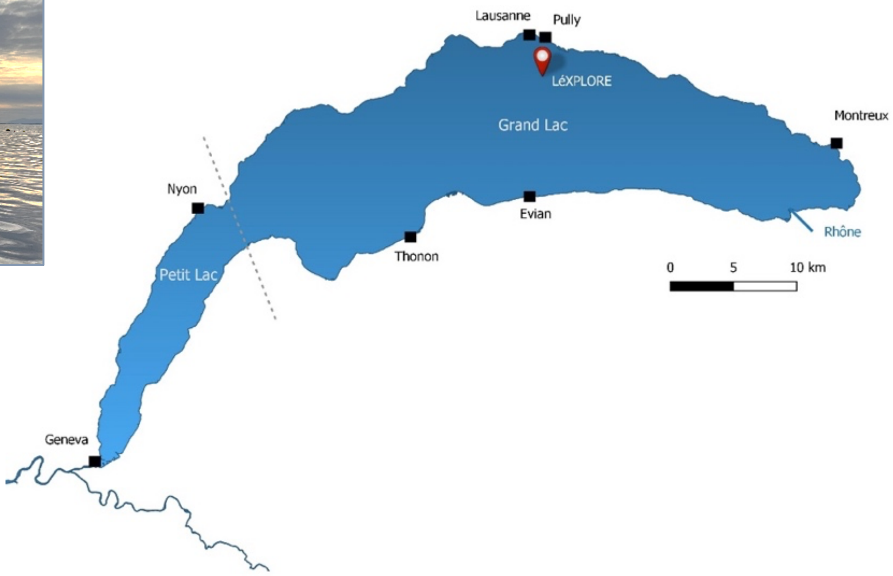

Fig. S2. Seasonal variations of environmental variables measured between August 2019 and December 2020. The small circles represent the actual measures detected in the water samples while colors represent smooth approximations of the measures between dates and depths determined using local polynomial regression fitting.

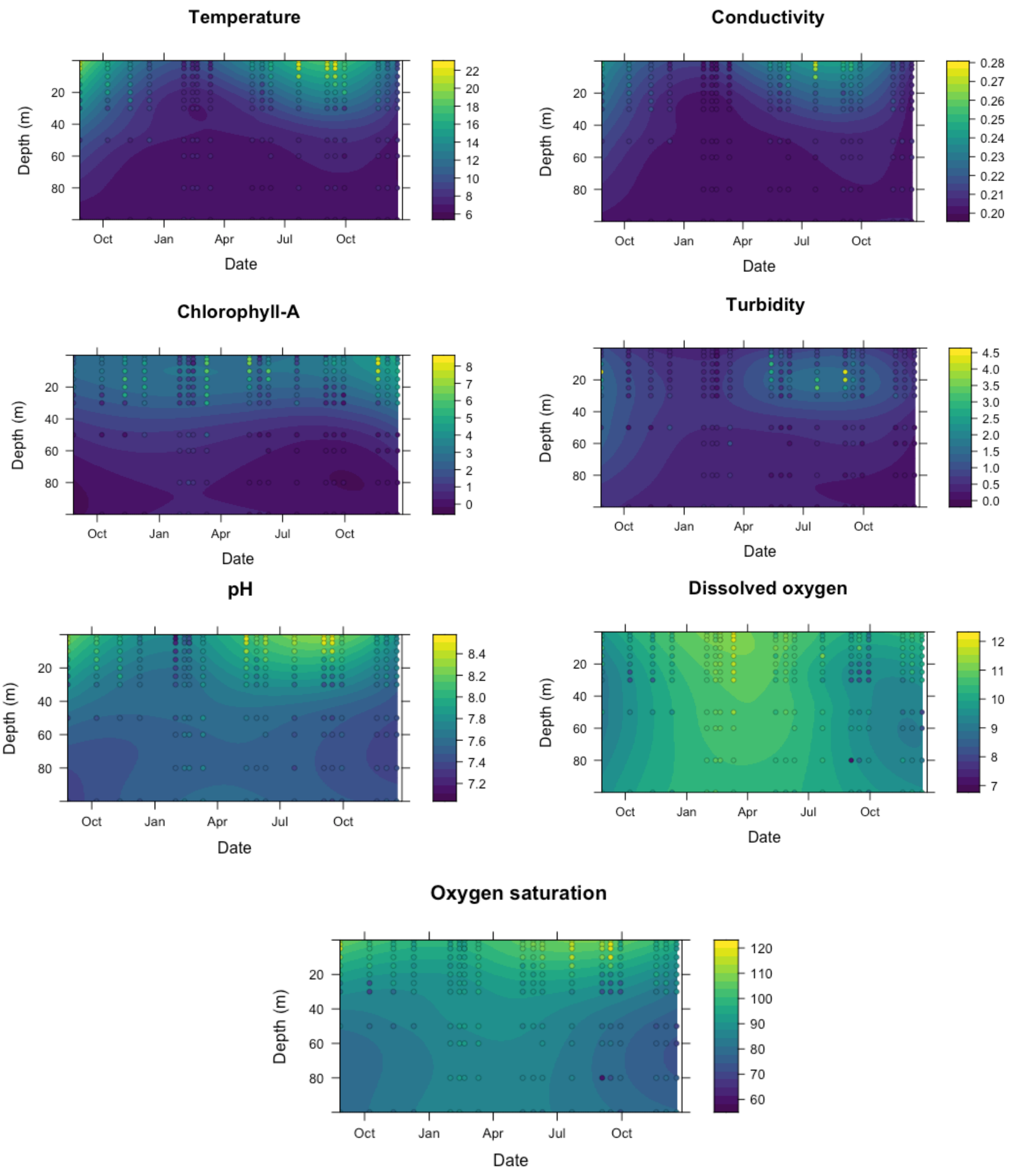

Fig. S3. Seasonal variations of nutrient concentrations measured between August 2019 and December 2020 through the water column. The units of these measures correspond to µg/l. The small circles represent the actual measures detected in the water samples while colors represent smooth approximations of the measures between dates and depths determined using local polynomial regression fitting.

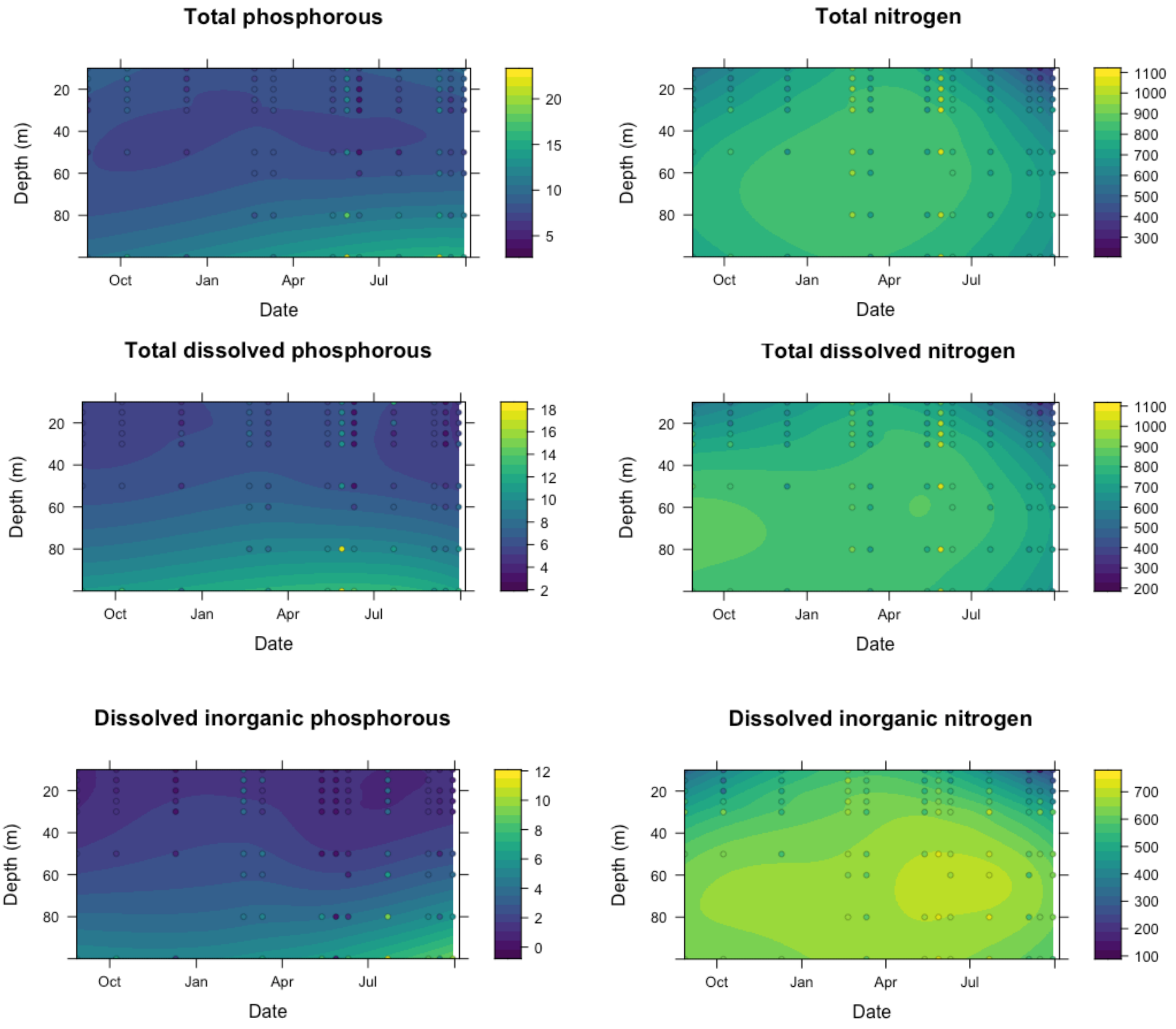

Fig. S4. Nonmetric Multidimensional Scaling (NMDS) analysis based on Bray-Curtis dissimilarity distances. Samples are colored by layer in the water column (A) and by season (B).

A. Layer

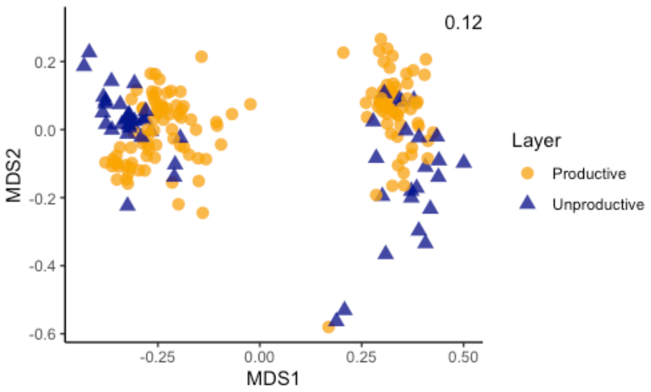

B. Season

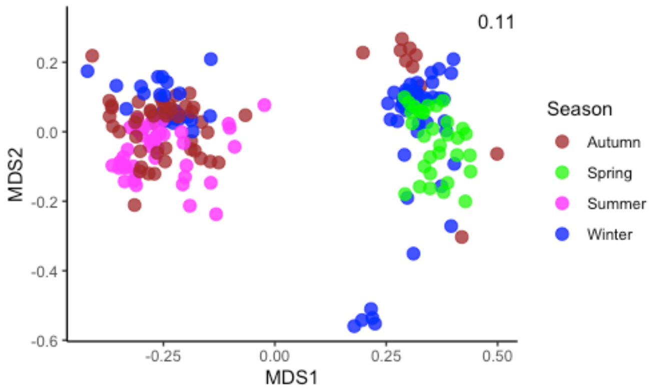

Fig. S5. Cyanobacteria community composition. Main cyanobacteria species identified in the dataset.

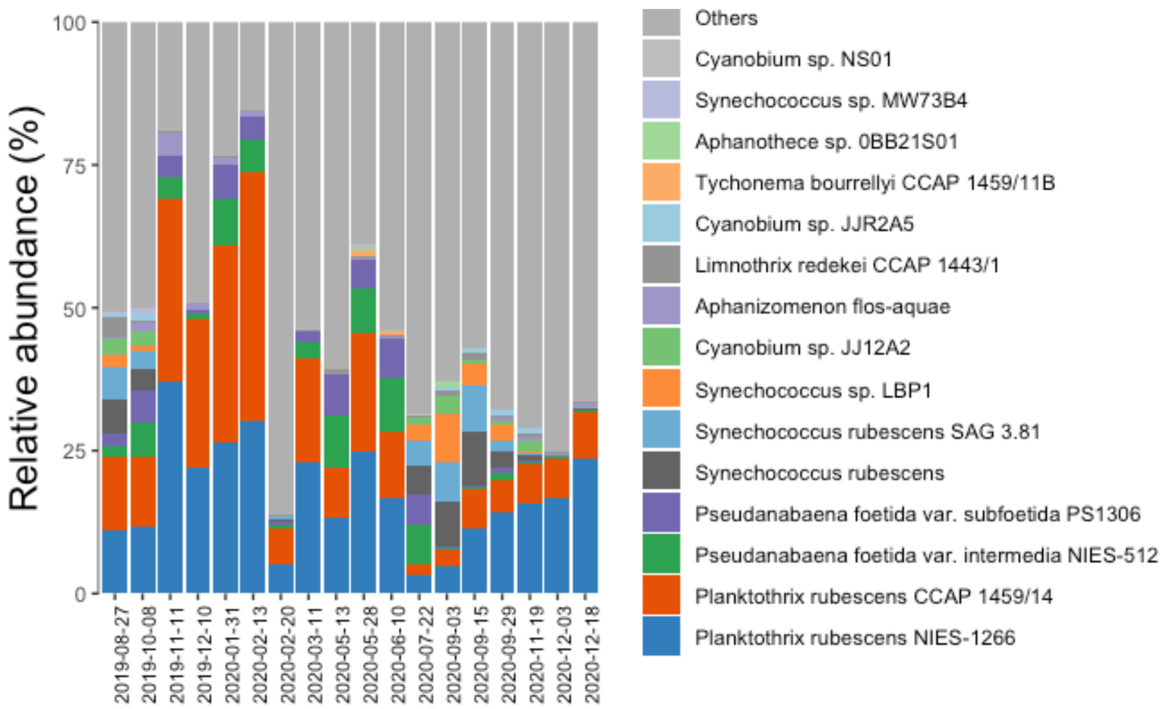

Fig. S6. RDA analyses of the environmental conditions of the study by sampling dates.

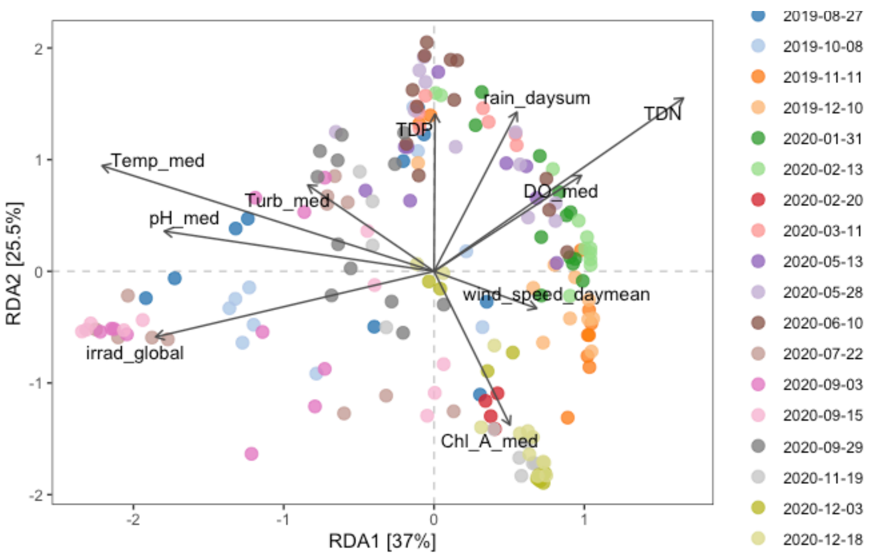

Fig. S7 Bacterioplankton predicted biomarkers in Lake Geneva. Top LEfSe-predicted taxonomic and functional biomarkers for the top (0-30 m) and bottom (30-100 m) layers in the water column.

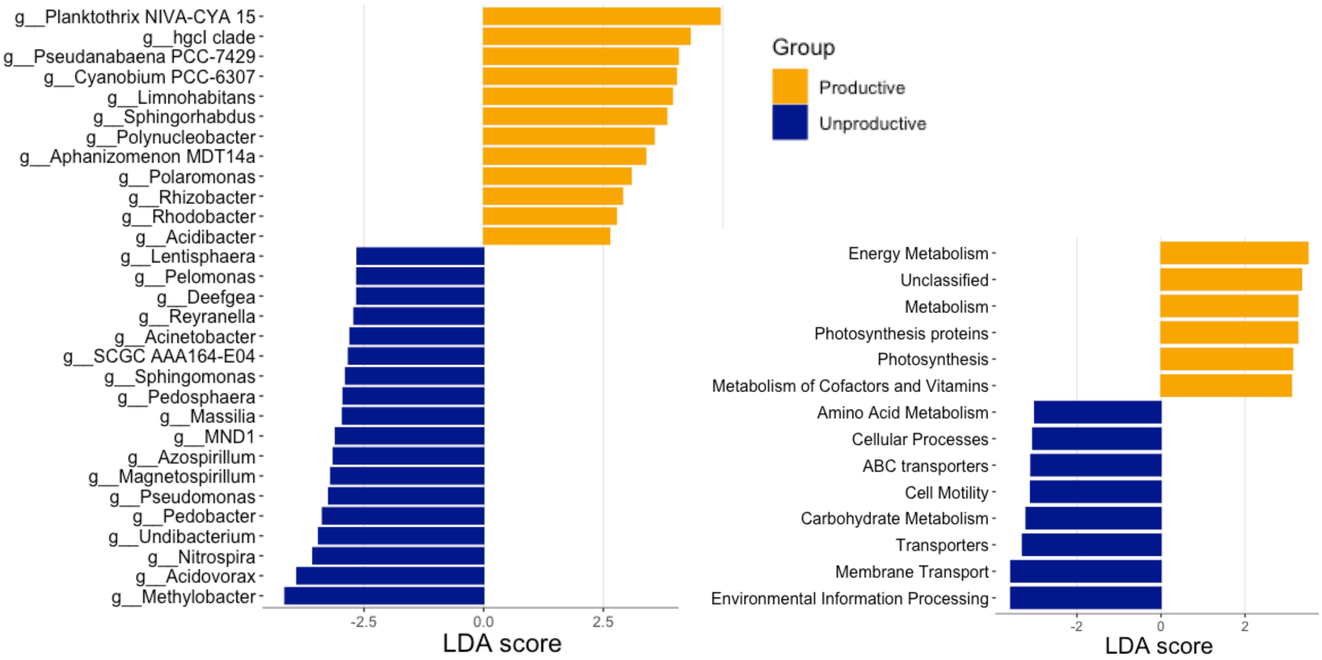

Fig. S8. Taxonomic biomarkers for the different seasons obtained using Lefse algorithm based on KEGG pathway predictions according to Picrust2 predictions. LDA stands for linear discriminant analyses.

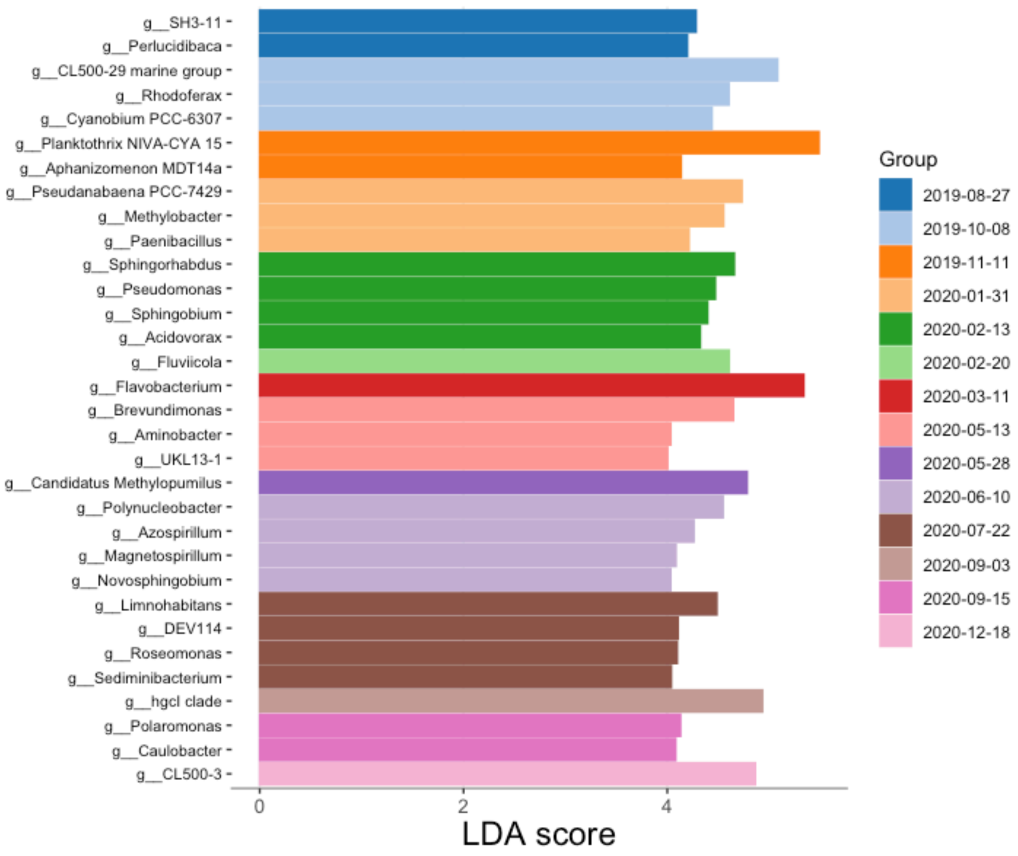

Fig. S9. Functional diversity plotted against taxonomic diversity and colored according to the date of sampling. Both diversities were estimated based on Chao1 indices.

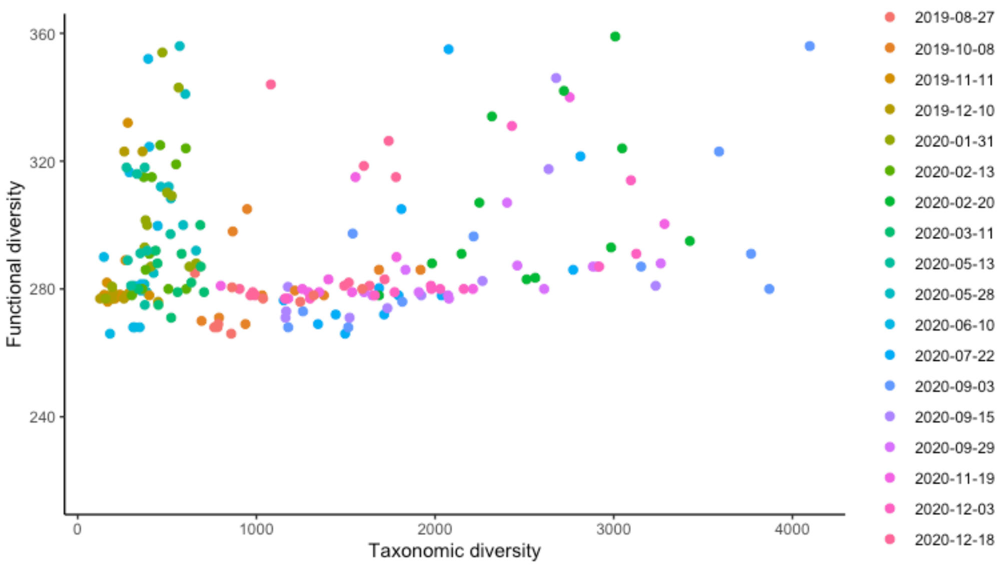

Fig. S10. Correlation matrix based on Spearman between the selected environmental variables and the taxonomic diversity indices. Irrad_global: Global solar irradiation. Temp_med: Water temperature. TDP: Total dissolved phosphorous. Chl_A_med: Chlorophyll A. Rain_daysum: Rain (as day sum). DO_med: Dissolved oxygen. Ph_med: pH. TDN: Total dissolved nitrogen. Turb_med: Turbidity. Wind_speed_daymean: Wind speed (as day mean).

A. Taxonomic diversity indices

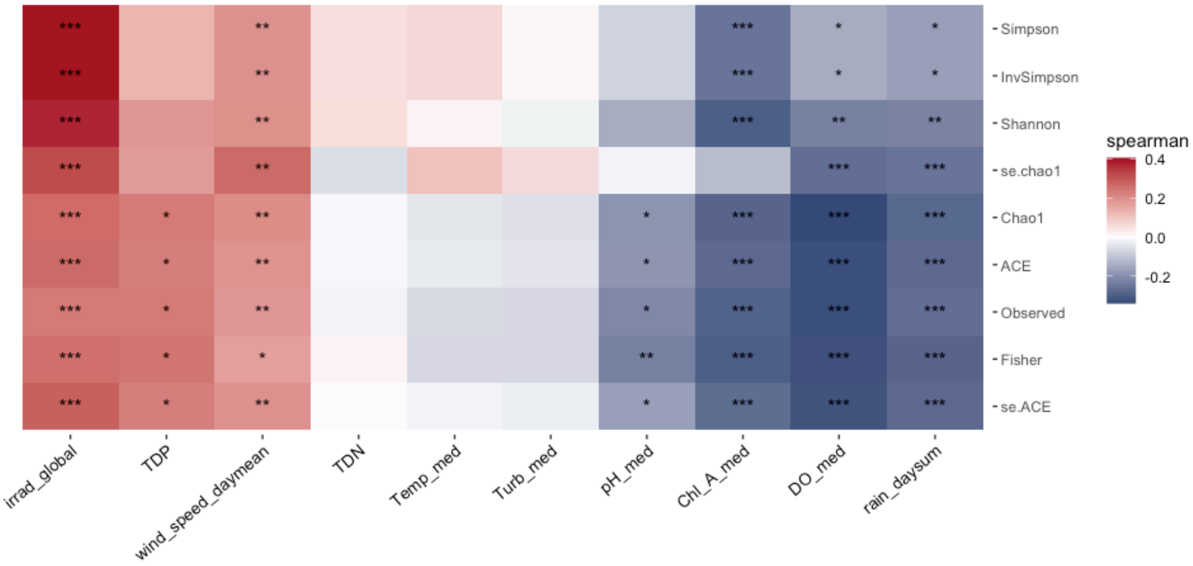

B. Functional diversity indices

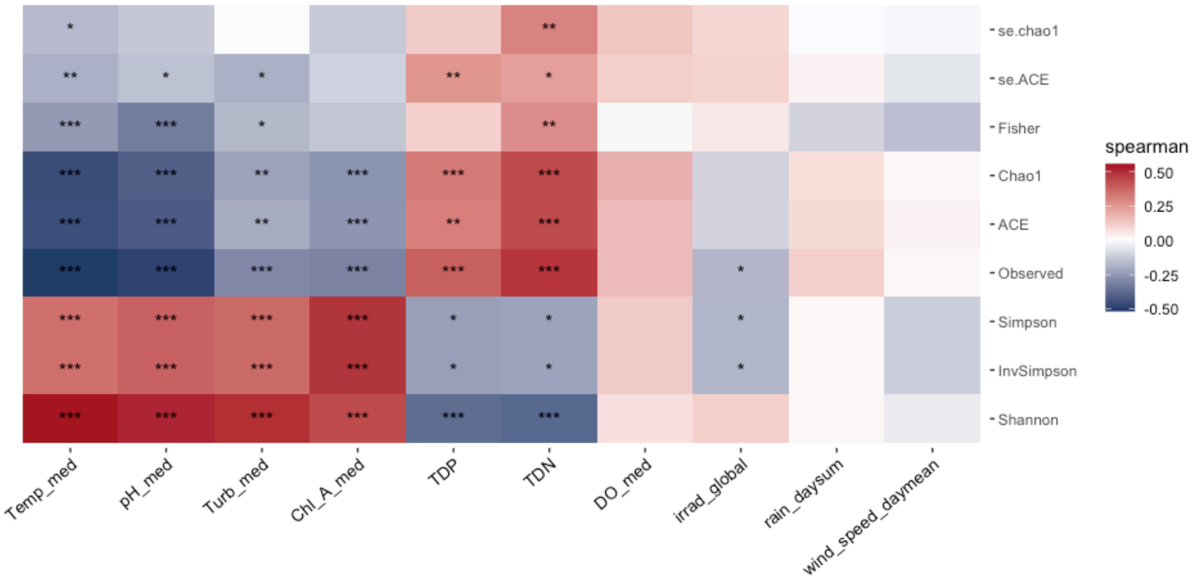

Supplement: Supplementary file 1 [file Image_1.pdf]
